# Virome and Experimental Analysis Reveal Tryptophan‐Like Dissolved Organic Matter Contributes to the Persistence of Plant Viruses in River Water

**DOI:** 10.1002/advs.202417529

**Published:** 2025-05-08

**Authors:** Yujie Wang, Ming Chen, Liu Yang, Jun Ma, Jian Tang, Shengjun Wu, Chi He, J. Paul Chen

**Affiliations:** ^1^ State Key Laboratory of Lake and Watershed Science for Water Security Chongqing Institute of Green and Intelligent Technology Chinese Academy of Sciences Chongqing 400714 China; ^2^ Chongqing School University of Chinese Academy of Sciences Chongqing 400714 China; ^3^ State Key Laboratory of Multiphase Flow in Power Engineering School of Energy and Power Engineering Xi'an Jiaotong University Xi'an 710049 China; ^4^ Department of Civil and Environmental Engineering National University of Singapore 10 Kent Ridge Singapore 117576 Singapore; ^5^ College of Chemistry and Environmental Engineering Shenzhen University Shenzhen 518060 China

**Keywords:** dissolved organic matter, environmental risks, persistence, plant viruses, virome

## Abstract

Plant viruses are devastating plant pathogens, resulting in annual losses exceeding billions of dollars in global agriculture. Once outside their plant host and insect vectors, plant viruses encounter unfavorable environmental factors that can accelerate their decay. However, plant viruses have been observed to persist in aquatic environment. The reasons why many plant viruses remain stable and infective in aquatic environment for extended periods are still largely unknown. In this study, a virome approach is utilized to examine the presence of plant viruses in river water. The results indicated that tryptophan‐like dissolved organic matter (Try‐like DOM) may play a crucial role influencing the abundance of plant viruses in river water. Further experiments found that Try‐like DOM can protect plant viruses under simulated natural conditions. This protective phenomenon is attributed to Try‐like DOM adopting a “swimming firewall mode”, i.e., free Try‐like DOM can act as a firewall to effectively absorb ultraviolet (UV) light, helping plant viruses avoid the harmful effects of UV radiation and free radicals. These findings offer new insights into the mechanisms underlying the persistence of plant viruses in aquatic environment, which would be helpful in developing strategies to control the spread of plant viruses.

## Graphical abstract

Tryptophan‐like dissolved organic matter (Try‐like DOM), a key component of organic matter in river ecosystems, demonstrates significant protective effects against plant viruses. The protective mechanism operates via a “swimming firewall” mode, whereby free Try‐like DOM attenuates ultraviolet (UV) radiation and scavenges reactive oxygen species, thereby shielding plant viruses and enhancing their persistence in aquatic ecosystems.

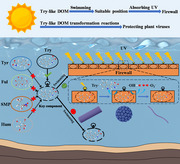

## 1 Introduction

Plant viruses account for nearly half of the pathogens that cause plant diseases, resulting in global annual yield losses estimated at over $30 billion.^[^ ^1^ ^]^ Many plant viruses infect staple crops and induce severe disease symptoms.^[^ ^2^ ^]^ Epidemics of viral diseases can significantly decrease food supplies, which may ultimately trigger large‐scale famine.^[^ ^3^ ^]^ Most plant viruses have limited mobility and rely on vectors (such as insects, nematodes, fungi, and water) to spread from one plant to another.^[^ ^4^ ^]^ Compared to insect‐ and fungus‐mediated transmission, waterborne transmission enables plant viruses to spread over longer distances.^[^ ^5^ ^]^

Aquatic environments serve as important reservoirs for the survival and transmission of plant viruses.^[^ ^6^ ^]^ Plant viruses are readily released from infected roots, injured or decaying plant material, and domestic sewage into water bodies.^[^ ^7^, ^8^ ^]^ Since plant viruses were reported in water bodies in the 1980s, they have been observed to be prevalent and abundant in various water bodies.^[^ ^9^, ^10^ ^]^ To date, plant viruses from at least seven different genera (Necroviruses, Diathoviruses, Potexviruses, Cucumoviruses, Carmoviruses, Tobamoviruses, and Tombusviruses) have been detected in streams, rivers, lakes, and oceans.^[^ ^11^ ^]^ Viral morphology provides the basis for grouping plant viruses.^[^ ^12^ ^]^ They are mainly classified into spherical, rod‐like, and filamentous plant viruses.^[^ ^13^, ^14^ ^]^ Among them, the rod‐shaped pepper mild mottle virus (PMMoV) has a global detection rate of 87% in surface freshwater, with viral concentrations detected as high as 7.4 log10 copies L^−1^ in river water.^[^ ^15^, ^16^ ^]^ Besides, the rod‐shaped cucumber green mottle mosaic virus recovered from river water remain infective to host plants.^[^ ^17^ ^]^

Utilization of river water contaminated with plant viruses for agricultural irrigation would increase risk of viral infection in crops. These viruses can directly contact with their roots or leaves, causing large‐scale plant diseases and severe economic losses in agricultural areas.^[^ ^18^, ^19^ ^]^ At the same time, waterborne plant viruses are highly stable, enabling them to infect crops even at low concentrations.^[^ ^20^, ^21^ ^]^ This increases plant virus control costs and intensifies negative impacts on global food security. In particular, the Yangtze River basin is a typical agricultural region in China, cultivating a large variety of crops such as rice, wheat, maize, and cotton.^[^ ^22^ ^]^ ≈51% of the total water consumption of the Yangtze River is utilized for agricultural irrigation.^[^ ^23^ ^]^ Therefore, it is imperative to investigate the reasons why many plant viruses remain stable and infective in river water.

In this work, to investigate the above question, we conducted a comprehensive virome study in the Yangtze River, which identified a significant correlation between tryptophan‐like dissolved organic matter (Try‐like DOM) and plant virus abundance in river water. By investigating the interaction between plant viruses and various types of DOMs under simulated natural conditions, we further discovered the role of Try‐like DOM in safeguarding plant virus from ultraviolet (UV) radiation. Our study revealed that the protective phenomenon was attributable to the unique “swimming firewall mode” of Try‐like DOM. In this mode, free Try‐like DOM can act as a protective shield to effectively absorb UV light and avoid the harmful effects of free radicals for plant viruses. This helps plant viruses to maintain their integrity and infectivity in river water.

## 2 Results

### 2.1 A Significant Positive Correlation is Present Between Try‐Like DOM and Plant Viruses

**Figure 1:**
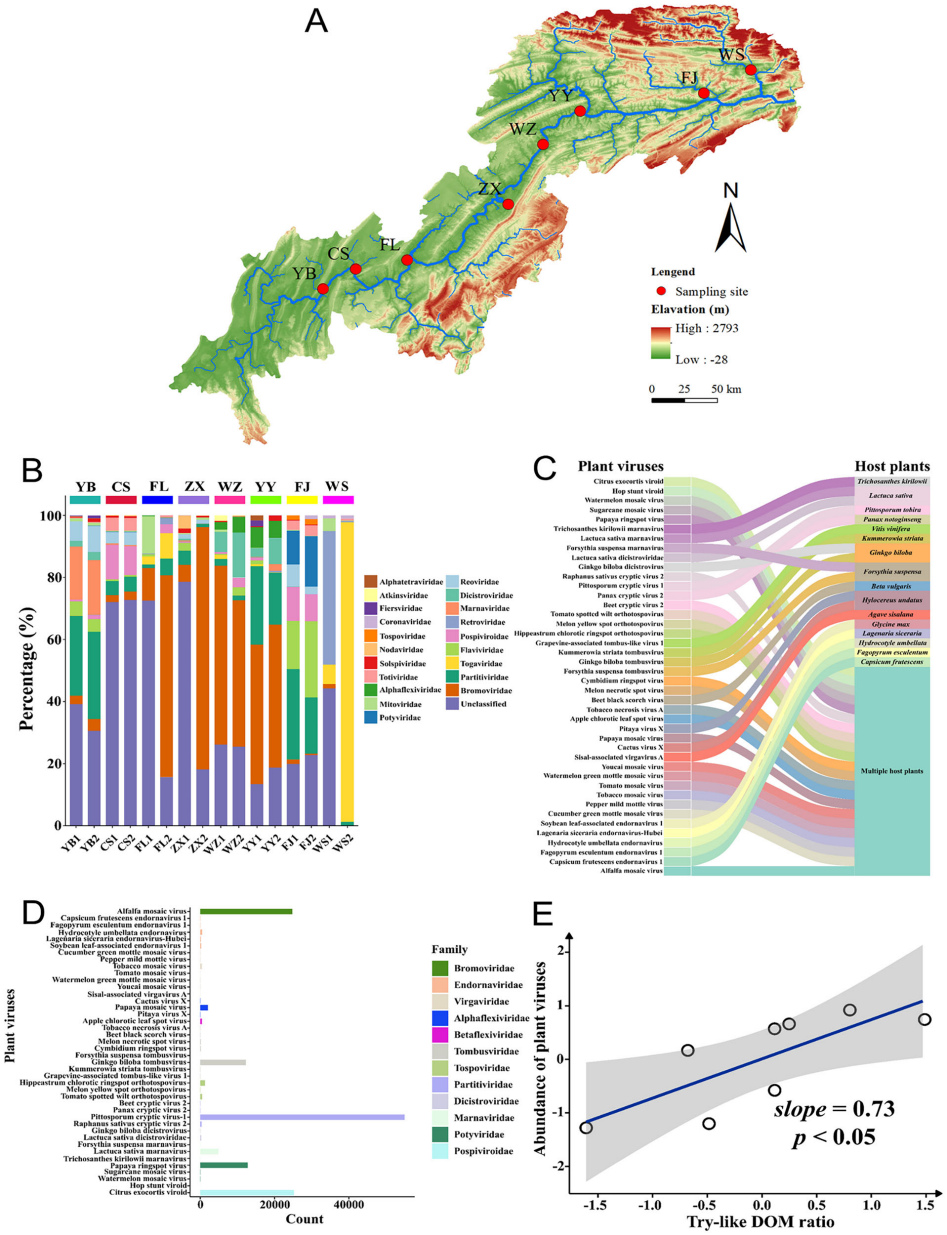
Identification of key contributors to influencing the persistence of plant viruses in river water. A) Map of sampling sites in the Yangtze River. B) The relative proportions of the top 20 RNA viral families normalized using reads per kilobase of transcript per million mapped reads (RPKM). C) Sankey diagram displaying the correspondence between plant viruses (left) and their host (right). D) The number of reads corresponding to contigs identified as plant viruses. E) The partial residual plot between the abundance of plant viruses and Try‐like DOM ratio analyzed by the best‐subset regression model (*n* = 8).

To systematically investigate the impact of environmental factors on plant viruses, we measured a variety of water environmental factors and employed the virome approach to detect plant viruses. We identified 42 plant virus species from 12 viral families by the virome approach in the Yangtze River (**Figure** **1**A,B). Among them, more than half of plant viruses were capable of infecting multiple host plants that included several species from one or more different plant families (Figure 1C and Text S1, Supporting Information). Compared to viruses associated with other host types, the plant viruses were found to be dominant (Figure S1, Supporting Information), counting 141 972 reads (Figure 1D and Table S1, Supporting Information). Their morphologies are mainly classified into three categories: spherical, rod‐like, and filamentous. The plant viruses constituted a significant proportion of RNA viruses in water samples, but the environmental factors that can prolong their persistence in river water is still unknown. To identify the factors significantly correlated with plant viruses, a subset regression was performed to determine the best‐fit model describing abundance of plant viruses. The combination of Try‐like DOM and soluble microbial products (SMP) outperformed to others (Table S2, Supporting Information). Notably, based on the optimal subset regression model, the abundance of plant viruses showed a significant positive correlation with Try‐like DOM (*slope* = 0.73, *p* < 0.05) (Figure 1E), indicating that the Try‐like DOM may play a vital role in facilitating the persistence of plant viruses in river water. In contrast, other factors did not show significant correlations with the abundance of plant viruses. The partial residual plot between the abundance of plant viruses and SMP ratio was presented (Figure S2A, Supporting Information). The linear regression plots between the abundance of plant viruses and other environmental factor were presented (Figure S2B–F, Supporting Information).

### 2.2 Try‐Like DOM has a Protective Effect on Plant Viruses in Water Under UV Radiation

DOM is ubiquitous in river water and can be classified into five distinct types based on their fluorescence characteristics.^[^ ^24^ ^]^ To investigate the role of Try‐like DOM on plant viruses, we simulated an aquatic environment based on the Yangtze River, which included factors such as UV radiation, pH, temperature and five types of DOMs. Moreover, the capsid protein (CP), as the first line of defence of plant viruses, plays an important role in preserving the integrity of viral genome and protecting it against environmental adversities.^[^ ^25^, ^26^ ^]^ When the structure of CP in plant viruses changes significantly, it commonly suggests the damage of plant viruses.^[^ ^27^, ^28^ ^]^ Thus, the CP of plant viruses frequently serves as a model for the study on the protective effect of plant viruses.^[^ ^29^ ^]^ Based on our virome data, we selected the three distinct morphologies of plant viruses for recombinantly expressing their CP: Pepper Mild Mottle Virus (PMMoV, rod‐shaped), Tomato Spotted Wilt Orthotospovirus (TSWV, spherical), and Papaya Ringspot Virus (PRSV, filamentous).^[^ ^30^, ^31^, ^32^ ^]^

**Figure 2:**
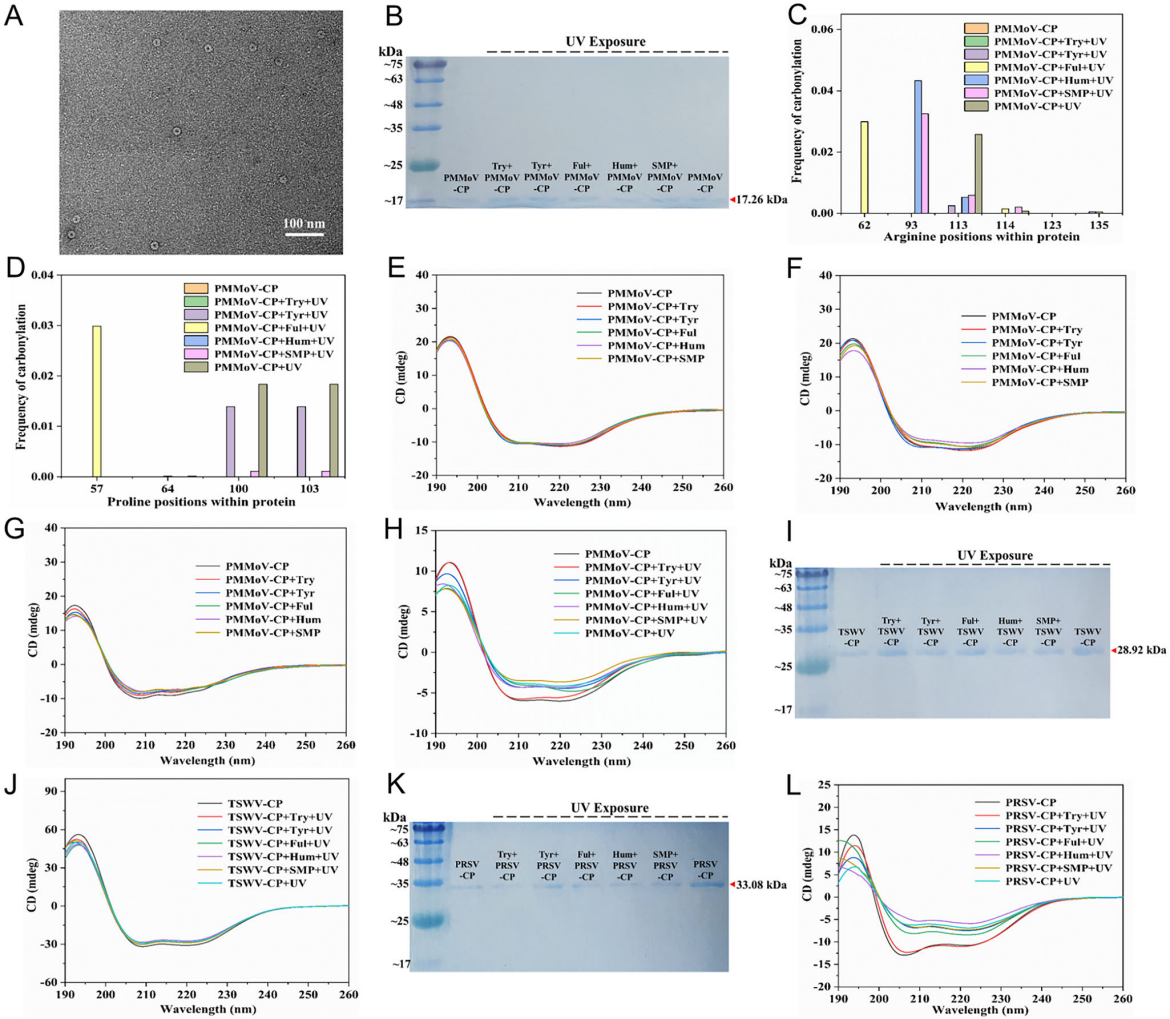
Effect of DOM on the primary and secondary structures of plant virus CP under varying conditions. A) Negative stain TEM image of PMMoV‐CP. B) SDS‐PAGE with Coomassie staining of PMMoV‐CP and PMMoV‐CP with different DOM systems after 12 h under UV radiation. Label‐free proteome analysis of carbonylated C) arginine and D) proline residues in PMMoV‐CP with different DOM systems after 12 h under UV radiation. E) CD spectra of PMMoV‐CP and PMMoV‐CP with different DOM systems. F) CD spectra of PMMoV‐CP and PMMoV‐CP with different DOM systems after 12 h at 18 °C. G) CD spectra of PMMoV‐CP and PMMoV‐CP with different DOM systems after 12 h at pH values of 8. H) CD spectra of PMMoV‐CP and PMMoV‐CP with different DOM systems after 12 h under UV radiation. I) SDS‐PAGE with Coomassie staining of TSWV‐CP and TSWV‐CP with different DOM systems after 12 h under UV radiation. J) CD spectra of TSWV‐CP and TSWV‐CP with different DOM systems after 12 h under UV radiation. K) SDS‐PAGE with Coomassie staining of PRSV‐CP and PRSV‐CP with different DOM systems after 12 h under UV radiation. L) CD spectra of PRSV‐CP and PRSV‐CP with different DOM systems after 12 h under UV radiation.

The SEC‐purified PMMoV‐CP was visualized by negative‐stain transmission electron microscope (TEM). The SEC‐purified PMMoV‐CP was ring‐like structures, which were composed of CP monomers (**Figure** **2**A). In sodium dodecyl sulfate ‐polyacrylamide gel electrophoresis (SDS‐PAGE) gel image, the molecular weight of PMMoV‐CP and PMMoV‐CP with individual DOM after UV radiation did not show obvious changes (Figure 2B). However, typical oxidative damage to protein (carbonylated arginine and proline) was detected by label‐free proteome in both PMMoV‐CP and PMMoV‐CP with different DOMs (tyrosine, Tyr; fulvic acid, Ful; humic acid, Hum and glucose, SMP) after UV radiation (9.5 mW cm^−2^) (Figure 2C,D). It is worth noting that the carbonylation of arginine and proline were not detected in PMMoV‐CP treated with Try when exposure to UV radiation. Moreover, circular dichroism (CD) spectroscopy was performed to further assess the changes of PMMoV‐CP in different treatment groups. When adding different DOMs to PMMoV‐CP, we did not observe significant changes in the CD spectra of PMMoV‐CP (Figure 2E). Besides, no significant alterations in the CD spectra of PMMoV‐CP were observed when PMMoV‐CP with different DOMs (Try, Tyr, Ful, Hum, and SMP) systems were placed at 18 °C and pH values of 8 conditions (Figure 2F,G). The significant alterations in the CD spectra were observed for PMMoV‐CP with different DOMs (Tyr, Ful, Hum, and SMP) after exposure to UV radiation (Figure 2H). No significant alterations in the CD spectra were observed for PMMoV‐CP with Try after exposure to UV radiation. (Figure 2H). The molecular weight and CD spectra of TSWV‐CP, as well as TSWV‐CP with individual DOM after UV radiation did not show obvious changes (Figure 2I,J). This observation is likely attributed to the intrinsic high stability of TSWV‐CP. The molecular weight of PRSV‐CP and PRSV‐CP with individual DOM after UV radiation did not show obvious changes (Figure 2K). The significant alterations in the CD spectra were observed for PRSV‐CP with different DOMs (Tyr, Ful, Hum, and SMP) after exposure to UV radiation (Figure 2L). No significant alterations in the CD spectra were observed for PRSV‐CP with Try after exposure to UV radiation. (Figure 2L).

We further analyzed the secondary structural content of these CP. When exposed to UV radiation, the content of α‐helix structure in both PMMoV‐CP and PRSV‐CP decreased significantly, except in systems where Try was added. (Figure S3, Supporting Information). Together, these results confirm that Try has a protective effect on the CP of plant viruses in water against the damaging UV radiation. Thus, Try‐like DOM can protect plant viruses from UV‐induced damage in river water by safeguarding their first line of defence.

### 2.3 Try‐Like DOM Protects Plant Viruses by a “Swimming Firewall Mode”

#### 2.3.1 Try‐Like DOM Protects Plant Viruses by Acting as a Firewall Under UV Radiation

**Figure 3:**
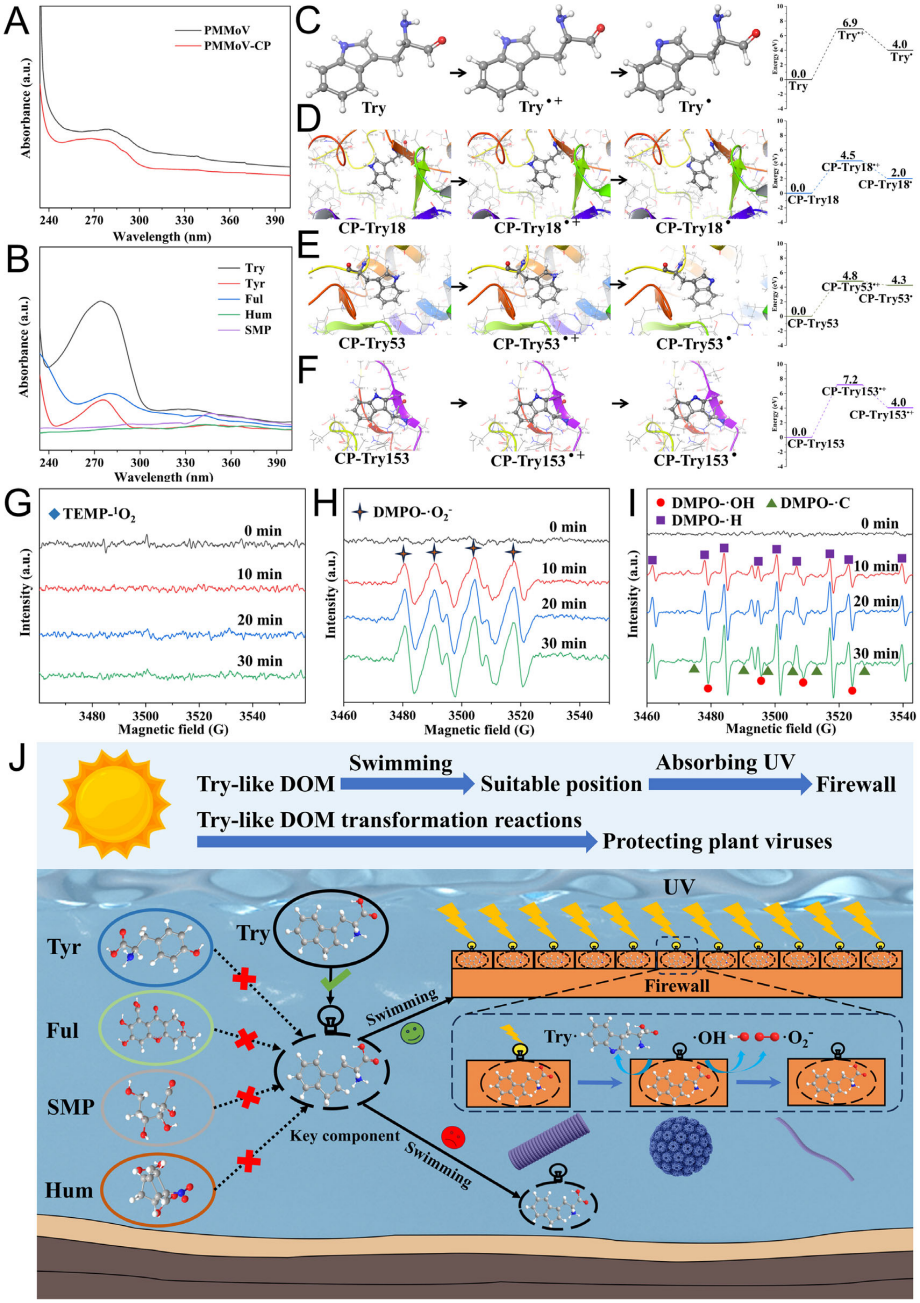
Mechanism of Try protecting plant viruses by a “swimming firewall mode”. A) UV–vis absorption spectra of PMMoV and PMMoV‐CP. B) UV–vis absorption spectra of different DOMs (Try, Tyr, Ful, Hum, and SMP). QM/MM energy profiles for the reaction process of Try at different states (energies are given relative), including C) free Try, D) Try at position 18 in PMMoV‐CP, E) Try at position 53 in PMMoV‐CP, F) Try at position 153 in PMMoV‐CP. G) TEMP spin‐trapping EPR spectra of Try under UV radiation in ultrapure water. H) DMPO spin‐trapping EPR spectra of Try under UV radiation in methanol. I) DMPO spin‐trapping EPR spectra of Try under UV radiation in ultrapure water. J) Schematic illustration of the protective mechanism of Try for plant viruses by a “swimming firewall mode.”.

To better understand the key protection mechanisms, UV–vis absorption spectra, quantum mechanics/molecular mechanics (QM/MM) energy calculations, electron paramagnetic resonance (EPR) spectroscopy and liquid chromatograph mass spectrometer (LC‐MS) analysis were performed. UV–Visible absorption spectra were utilized to assess the UV‐absorbing capacity of PMMoV, PMMoV‐CP, and Try. The absorption spectra of PMMoV and PMMoV‐CP displayed a single absorption peak in the range of 262–309 nm and 249–310 nm, respectively (**Figure** **3**A). The absorption spectra of Try exhibited a broader and more pronounced absorption peak in the range of 239–312 nm (Figure 3B). Furthermore, we conducted QM/MM calculations to determine the reaction energy for Try in different states. We first equilibrated the PMMoV‐CP and Try system by molecular dynamics (MD) simulations. Our MD simulations showed that PMMoV‐CP underwent no significant conformational changes and exhibited only rare interactions with Try (Figure S4A–C, Supporting Information). QM/MM calculations showed that the energy barrier to reach the transition state (Try**^·+^**) from the free Try was 6.9 eV (Figure 3C). When Try was present at positions 18, 53, and 153 within the context of PMMoV‐CP, the corresponding energy barriers were 4.5, 4.8, and 7.2 eV, respectively (Figure 3D–F). The energy barrier for free Try is somewhat higher compared to Try at positions 18 and 53 within PMMoV‐CP, indicating that the protein environment (amino acid residues and interactions) can influence the reactivity of Try. The reaction free energy for free Try or Try within PMMoV‐CP was positive, indicating that the reaction cannot occur spontaneously. Moreover, through an investigation of Try at various depths, it has been discovered that the Try layer positioned above PMMoV is essential to its UV protection mechanism (Figure S5, Supporting Information). Therefore, the presence of Try can act as a firewall to avoid damaging Try within viral CP under UV radiation.

The potential generation of ^1^O_2_ and free radical in the reaction system was trapped by spin‐trapping agent, such as 2,2,6,6‐Tetramethyl‐4‐piperidone (TEMP) and 5,5‐dimethyl‐1‐pyrroline N‐oxide (DMPO), at various timepoints under UV radiation, and subsequently examined by EPR spectroscopy. After irradiation, no characteristic triplet line peaks of TEMP‐^1^O_2_ were detected (Figure 3G). Notably, the characteristic six‐line peaks of DMPO‐·O_2_^−^ were observed, and the corresponding peak intensity increased over time (Figure 3H). Besides, the EPR peak of DMPO‐·OH, DMPO‐·C, and DMPO‐·H were detected in the reaction system, and their corresponding peak intensities also increased over time (Figure 3I). To further clarify the reaction pathways, LC‐MS were applied to determine the intermediates of Try after UV radiation. Three reaction pathways for Try to absorb UV light were proposed and illustrated (Figure S6, Supporting Information). Initially, Try absorbing UV light to form singlet‐state Try (^1^Try) and triplet‐state Try (^3^Try). In ·C‐mediated pathway, an electron can be ejected from the indole ring due to the high reactivity of ^3^Try, leading to the formation of a Try radical cation (Try^·+^). Subsequently, Try^·+^ can rapidly deprotonate to the neutral Try radical (Try^·^) (Figure S7, Supporting Information). Try^·^ can react with molecular oxygen to form a ring C‐3 peroxyl radical (P4), which can undergo further hydrogen atom abstraction reactions (Figure S7, Supporting Information). On the other hand, ^3^Try can transfer energy to oxygen and water molecules, leading to the generation of ·O_2_^−^ and ·OH, which in turn oxidizes Try. In ·OH‐mediated pathway, the intermediates (P9, P10, and P11) were generated through a series of cyclization reactions (Figure S7, Supporting Information). Moreover, ·O_2_^−^ can undergo rapid cycloaddition reactions with Try to the formation of cyclic peroxides (P5 and P6) (Figure S7, Supporting Information). These intermediates were likely to undergo further conversion into N‐formylkynurenine and N‐Formylanthranilic acid (P7 and P8) (Figure S7, Supporting Information). Therefore, Try acts as a firewall for plant viruses, effectively absorbing UV light energy and leading to the formation of short‐lived free radicals and reactive intermediates (Figure 3J). This firewall mechanism helped plant viruses to neutralize the harmful effects of UV radiation, thereby contributing to their persistence in river water.

#### 2.3.2 Try‐Like DOM and Plant Viruses are in a Swimming State

**Figure 4:**
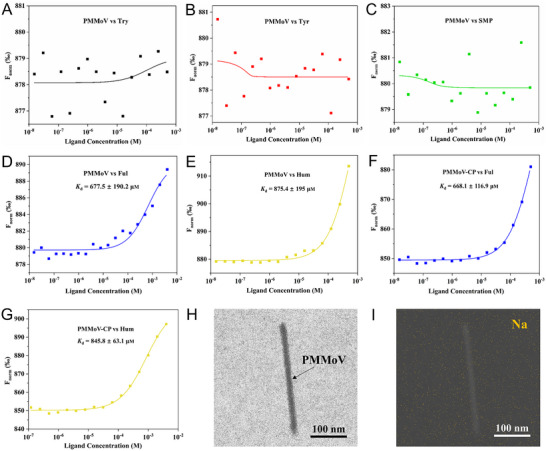
Binding affinities of DOM for PMMoV and PMMoV‐CP. Dose–response curve for the binding interaction between A) PMMoV and Try, B) PMMoV and Tyr, C) PMMoV and SMP, D) PMMoV and Ful, E) PMMoV and Hum, F) PMMoV‐CP and Ful, G) PMMoV‐CP and Hum. H) TEM image showing the mixture of Try‐Na and PMMoV. I) Corresponding TEM elemental mapping image of Na distribution.

If Try‐like DOM effectively binds to plant viruses, the generation of free radicals from Try‐like DOM absorbing UV radiation can cause damage to plant viruses. Therefore, we initially hypothesized that Try‐like DOM is in a swimming state to protect plant viruses. When Try‐like DOM swims above plant virus, Try‐like DOM can effectively block UV radiation damaging plant viruses. To confirm the interaction between DOM and plant virus, we analyzed the binding affinities of different DOMs with model plant viruses (PMMoV) using a microscale thermophoresis (MST) assay with fluorescent labeling. Our results showed no significant changes in the thermophoresis signal, indicating a weak or no affinity between Try and PMMoV (**Figure** **4**A). Likewise, we observed no significant change in dose‐response curves when altering the concentrations of Tyr and SMP, indicating that binding was little or not taking place (Figure 4B,C). Remarkably, when PMMoV was titrated with increasing concentrations of Ful and Hum, we could observe a saturable signal increase that could be fitted to a dissociation constant (*K*_d_) value of 677.5 ± 190.2 µM and 875.4 ± 195 µM, respectively (Figure 4D,E).

To elucidate origins of the observed binding affinities, Ful and Hum were used to titrate PMMoV‐CP. The *K*_d_ value of Ful and Hum binding PMMoV‐CP were determined to be 668.1 ± 116.9 µM and 845.8 ± 63.1 µM, respectively (Figure 4F,G). Significantly, the *K*_d_ values for the binding of Ful and Hum to both PMMoV‐CP and PMMoV were similar. The results demonstrated that Ful and Hum can bind to PMMoV through PMMoV‐CP, but their affinity toward PMMoV was low.

To further investigate the interaction between Try and PMMoV, TEM was used to directly observe the mixture of sodium‐modified Try (Try‐Na) and PMMoV. Try‐Na formed stable complexes through tridentate binding to the carbonyl oxygen atom, the amino nitrogen atom, and the π cloud of the aromatic ring (Figure S8, Supporting Information). TEM imaging revealed that PMMoV exhibited a rod‐shaped morphology, with approximate length of 300 nm and a width of 18 nm (Figure 4H). Moreover, the corresponding elemental mapping results also showed the homogeneous distribution of Na elements in the presence of both Try‐Na and PMMoV, suggesting a lack of significant affinity between Try and PMMoV (Figure 4I). Overall, our results suggest that Try‐like DOM is in a swimming state, providing protection for plant viruses. This ensures that the generation of free radicals from Try‐like DOM absorbing UV radiation cannot directly damage plant viruses, owing to the significant distance between the free radicals and plant viruses.

### 2.4 Try‐Like DOM can Preserve the Integrity and Infectivity of Plant Viruses

**Figure 5:**
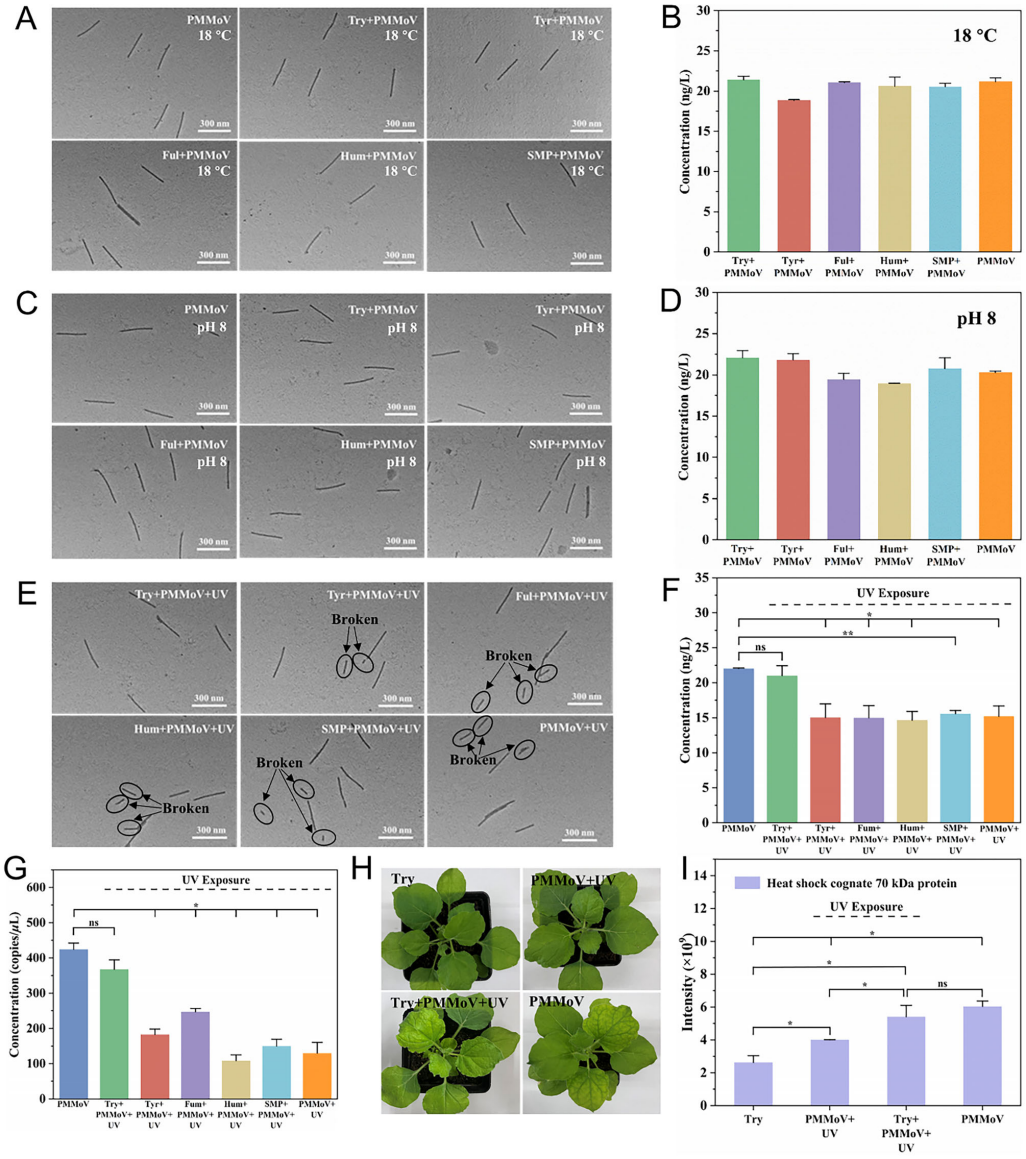
Effect of DOM on PMMoV under different environmental conditions. A) TEM images of PMMoV and PMMoV with different DOM systems after 12 h at 18 °C. B) ELISA measurements of surface antigens levels in PMMoV and PMMoV with different DOM systems after 12 h at 18 °C (*n* = 2). C) TEM images of PMMoV and PMMoV with different DOM systems after 12 h at pH values of 8. D) ELISA measurements of surface antigens levels in PMMoV and PMMoV with different DOM systems after 12 h at pH values of 8 (*n* = 2). E) TEM images of PMMoV and PMMoV with different DOM systems after 12 h under UV radiation. F) ELISA measurements of surface antigens levels in PMMoV and PMMoV with different DOM systems after 12 h under UV radiation (two‐sample *t*‐test, *n* = 2). G) RT‐qPCR measurement of PMMoV‐CP gene levels in PMMoV and PMMoV with different DOM systems after 12 h under UV radiation (two‐sample *t*‐test, *n* = 3). H) Symptoms of *Nicotiana benthamiana* inoculating Try, untreated PMMoV, PMMoV, and PMMoV with Try after UV radiated. Photographs were taken at 21 days post‐inoculation (dpi). I) The intensity of heat shock cognate 70 kDa protein in *Nicotiana benthamiana* determined by PRM‐MS (two‐sample *t*‐test, *n* = 2). Asterisks indicate significant difference (*p ≤ 0.05, **p ≤ 0.01). ns indicates not significant difference.

To confirm the role of different DOMs in plant viruses, the TEM and the enzyme‐linked immunosorbent assay (ELISA) were employed to study the morphology and surface antigens of the model plant viruses under different environmental conditions, including temperature, pH, and UV radiation. Our results illustrated that there was no significant difference in the size and shape of PMMoV when exposed to simulated natural temperature and pH conditions (**Figure** **5**A,C). The presence of DOM in the water did not affect the morphology of PMMoV. Furthermore, the surface antigens of PMMoV remained relatively stable across different types of DOMs under simulated natural temperature and pH conditions (Figure 5B,D).

In contrast, upon exposure to simulated natural UV radiation, a distinct morphological break in PMMoV was observed. (Figure 5E). The addition of Try‐like DOM can protect plant viruses from UV radiation affecting the morphology of PMMoV by adopting a “swimming firewall mode”. The average concentration of untreated PMMoV stock measured by ELISA was found to be 22 ng L^−1^, which was higher than both PMMoV stock and PMMoV stock with DOMs (Tyr, Ful, Hum, and SMP) after UV radiation (two‐sample *t*‐test, *p* < 0.05). Interestingly, the concentrations of PMMoV stock with Try after UV radiation did not differ significantly from those measured in untreated PMMoV stock (two‐sample *t*‐test, *p* > 0.05). The results of ELISA confirmed that Try provided protection against surface antigens of PMMoV under UV radiation (Figure 5F). To further investigate whether Try could also offer protection to the nucleic acid of PMMoV, real‐time quantitative reverse transcription polymerase chain reaction (RT‐qPCR) assays were employed. The absolute concentration of PMMoV‐CP gene in untreated PMMoV stock showed a significant difference compared to both PMMoV stock and PMMoV stock with different DOMs (Tyr, Ful, Hum, and SMP) after UV radiation (two‐sample *t*‐test, *p* < 0.05) (Figure 5G). The absolute concentration of PMMoV‐CP gene in between untreated PMMoV stock and PMMoV with Try after UV radiation did not show a significant difference (two‐sample *t*‐test, *p* > 0.05).

To evaluate the infectivity of PMMoV with Try after UV radiation, a series of samples were inoculated into the model plant (*Nicotiana benthamiana*) (Figure 5H). *Nicotiana benthamiana* plants inoculated with PMMoV stock after UV radiation and Try solution did not develop observable symptoms. In contrast, characteristic viral infection symptoms (leaf chlorosis) appeared when *Nicotiana benthamiana* plants were inoculated with either untreated PMMoV stock or PMMoV stock adding Try after UV radiation, respectively. Similar results were obtained in experiments conducted using peppers (*Capsicum annuum* L.) (Figure S9, Supporting Information). Additionally, we utilized parallel reaction monitoring‐mass spectrometry (PRM‐MS) to quantify the levels of heat shock cognate 70 kDa protein, which is commonly observed to accumulate in *Nicotiana benthamiana* infected with plant viruses.^[^ ^33^ ^]^ As a result, the peptide intensities of the heat shock cognate 70 kDa protein showed a significant difference in *Nicotiana benthamiana* inoculated with untreated PMMoV stock compared to PMMoV stock treated with UV radiation (Figure 5I). Notably, no significant difference in peptide intensities were detected in *Nicotiana benthamiana* when inoculated with untreated PMMoV stock and PMMoV stock adding Try after UV radiation. These results clearly demonstrated that Try has a protective effect against UV radiation on the nucleic acid and CP of plant viruses. Consequently, the presence of Try‐like DOM in river water can sustain their integrity and infectivity.

## 3 Discussion

Waterborne transmission has long been an overlooked route for the spread of plant viruses, largely because of technological limitations in viral detection. Recent technical advancements, particularly the application of high‐throughput sequencing in virome, have greatly improved the ability to characterize plant viruses in environmental water samples. While current virome studies have predominantly focused on elucidating the diversity and distribution of plant viruses, emerging evidence suggests that various environmental factors play crucial roles in viral persistence and inactivation.^[^ ^34^, ^35^ ^]^ However, it remains uncertain which specific environmental factors in river water, and by what mechanisms, significantly contribute to the prolonged persistence and sustained infectivity of plant viruses.

Here, to identify the key contributor among numerous potential environmental factors influencing the spread of plant viruses, we analyzed 16 samples collected from the Yangtze River using meta‐transcriptomics. Plant viruses were found to be the dominant RNA viruses in these samples, belonging to 42 species from 12 viral families. Most of these plant viruses have a wide range of plants as hosts. The broad host range of plant viruses allows them to exploit various ecological niches and infect suitable hosts in diverse habitats. Furthermore, plant viruses can enter river water in different ways.

Plant viruses can spread over long distances and remain stable in aquatic environments, despite having lower infection efficiency than insect‐, fungi‐, and nematode‐borne plant viruses.^[^ ^36^ ^]^ The persistence of plant viruses in river water can be affected by not only the stability of themselves, but also their interaction with various environmental factors.^[^ ^37^ ^]^ DOM is a complex mixture of organic compounds that is ubiquitously present in river water and controls light attenuation.^[^ ^38^ ^]^ DOM not only participates actively in various biochemical processes but also exerts a notable influence on the migration and transformation of microorganisms.^[^ ^39^ ^]^

Our study found that Try‐like DOM is a key factor to the spreading of plant viruses in river water through virome and best‐subset regression analysis. Try‐like DOM represents an important component of organic matter in river ecosystems, which typically exhibits excitation peaks at wavelengths ≈220–230 nm and 270–280 nm, and emission peaks at ≈350 nm.^[^ ^40^ ^]^ Previous studies have indicated that Try‐like DOM is derived from soil and higher plants, making it more likely to encounter and interact with plant viruses.^[^ ^41^, ^42^, ^43^ ^]^ Moreover, in contrast to animal viruses, the majority of plant viruses lack an envelope and typically encode movement proteins that mediate the intercellular transport of virions through plasmodesmata.^[^ ^44^, ^45^ ^]^ The non‐enveloped structures of plant viruses expose their CP, which possess surfaces rich in charged residues.^[^ ^46^ ^]^ The surface‐exposed charged residues on plant viruses are likely to facilitate interfacial interactions with Try‐like DOM.^[^ ^47^ ^]^

To elucidate the relationship between plant viruses and Try‐like DOM, we selected typical plant viruses present in river water and recombinantly expressed their CP. A series of experiments were conducted under conditions that simulate the water environment of the Yangtze River. Oxidative damage in CP of plant viruses was determined by label‐free proteome.^[^ ^48^ ^]^ The significant structural changes were evaluated using CD spectroscopy.^[^ ^49^ ^]^ We observed that Try‐like DOM has a protective effect on plant viruses in water under UV radiation across various conditions. For the viruses with less stable capsids, the protective effects of Try‐like DOM are likely to be particularly pronounced. The protection of Try‐like DOM for plant viruses can enhance their survival and dissemination in river water, eventually leading to increased risks of viral infection for plants.

We further found that this protective effect can be attributed to Try‐like DOM adopting a “swimming firewall mode.” Try‐like DOM displays an outstanding capacity for absorbing and quenching UV light in water.^[^ ^50^ ^]^ Moreover, recent work suggested Try‐like DOM is highly active in maintaining the biological effectiveness in river water.^[^ ^51^ ^]^ Consequently, the presence of Try‐like DOM in river water acts as a firewall, effectively absorbing UV light and thereby reducing the penetration of UV radiation damage to plant viruses. In this mode, the generation of free radicals from Try‐like DOM absorbing UV radiation were difficult to damage plant viruses due to the swimming state of Try‐like DOM. Consistent with this idea, previous studies have indicated that electrostatic repulsion exists between viruses and DOM, significantly weakening their direct interaction.^[^ ^14^ ^]^ Therefore, Try‐like DOM crucially contributes to the spreading of plant viruses in river water by a “swimming firewall mode.”

In conclusion, our study has revealed that Try‐like DOM plays a role in enhancing the persistence of plant viruses in river water. First, we observed that Try‐like DOM has a significant correlation with plant viruses in river water. To further investigate this phenomenon, we conducted experiments that confirm the protective effect of Try‐like DOM for plant viruses against UV radiation via a “swimming firewall mode.” These insights enhance our understanding of how plant viruses persist in aquatic environment and may offer valuable implications for controlling plant viruses. Future research should consider the complex environmental factors influencing plant viruses and conduct long‐term monitoring in aquatic environments, which contribute to the development of risk assessment models and mitigation strategies for plant viruses.

## 4 Experimental Section

### Sample Collection and Analysis

Water samples were collected from the upper reaches of the Yangtze River in August 2022, covering Yubei District (YB), Changshou District (CS), Fuling District (FL), Zhong County (ZX), Wanzhou District (WZ), Yunyang County (YY), Fengjie County (FJ) and Wushan County (WS). The sampling sites span a geographic range from 29°66′N to 31°25′N and 106°88′E to 109°82′E. Meanwhile, water quality parameters were measured (Text S2, Supporting Information). Virome approach was used for the collection and analysis of viruses (Figure S10 and Text S3, Supporting Information).

### Inoculation and Extraction of PMMoV Particles

Seeds of wild‐type *Nicotiana benthamiana* were germinated in a pot filled with sterilized nutrient soil and vermiculite (1:1) substrate. The pot was then placed in an artificial climate chamber (HP1500GS‐LED, Ruihua, China) set at 25 °C, 60% relative humidity and 16/8 h light/dark cycle. After two weeks, the rooted plantlets were individually transferred to pots containing a substrate mixture, and a liquid fertilizer solution was sprayed on them every 2 days. When the plants reached the 4/5‐leaf stage, the leaves were inoculated by injecting purified virions of PMMoV using a needleless syringe. The leaves displaying viral symptoms were harvested three weeks after inoculation. PMMoV particles were subsequently purified from inoculated *Nicotiana benthamiana* leaves using Gooding's method.^[^ ^52^ ^]^ The purified PMMoV particles was stored in 0.01 mol L^−1^ phosphate buffer (pH 7.4) at −80 °C until further use.

### Expression and Purification of Plant Virus CP

To express PMMoV‐CP, the corresponding gene (GenBank accession No. KP955400.1) with PreScission protease cleavage site and 6 × His tag sequence at the N‐terminus was cloned into a pET28a vector. The recombinant plasmid was then transformed into expression host strain E. coli Rosetta (DE3) pLysS by heat shock treatment. Cells carrying the recombinant plasmid were grown in LB liquid medium containing 50 µg mL^−1^ kanamycin at 37 °C with shaking (220 rpm) until reaching OD600 = 0.6–0.8. The protein expression was induced by adding isopropyl β‐D‐thiogalactopyranoside (IPTG) with a final concentration of 0.1 mm overnight at 18 °C. The cells were collected with centrifugation at 4000 rpm for 15 min at 4 °C and used for subsequent purification. The collected cells were mixed with Ni‐Buffer A (20 mm Tris‐HCl, 150 mm NaCl, 20 mm imidazole, pH 7.4) at a ratio of 1:10 (1 g cells/10 mL buffer) and lysed by sonication in ice bath. Lysates were collected by centrifugation at 12 000 rpm for 30 min at 4 °C, loaded on Ni‐NTA columns equilibrated with Ni‐Buffer A and eluted using Ni‐Buffer B (20 mm Tris‐HCl, 150 mm NaCl, 500 mm imidazole, pH 7.4). The purified protein was cleaved by PreScission protease and loaded on size exclusion columns equilibrated with SEC‐Buffer (20 mm Tris, 150 mm NaCl, pH 7.4) for further purification. The high purity of PMMoV‐CP (>90%) was collected and stored at −80 °C. Moreover, TSWV‐CP and PRSV‐CP were expressed and purified as described in Text S4 (Supporting Information).

### Structural Analysis of CP

To evaluate changes in the primary and secondary structures of CP, SDS‐PAGE, and CD spectroscopy were performed. For SDS‐PAGE, a 12% running gel and 5% stacking gel were used. The CP samples were mixed with an equal volume of 4×loading buffer (P1016, Solarbio, China) and heated at 95 °C for 5 min. The CP samples were loaded, and electrophoresis was conducted on the sample at 150 V. Coomassie Brilliant Blue was used for protein identification. CD spectroscopic measurements were conducted using a circular dichroism spectrometer (JASCO 810, JASCO, Japan) at room temperature. Samples were loaded into quartz cuvette with a path‐length of 1 mm. Wavelength scans were performed from 400 to 190 nm at intervals of 0.5 nm. To analyze the data, the CD spectra of buffer were determined and subtracted from the CD spectra of samples. The CDNN software was used to calculate the secondary structure content of each sample.^[^ ^53^ ^]^

### ELISA

A double antibody sandwich ELISA kits (New Cell & Molecular Biotech, China) was used according to the manufacturer's instructions. Briefly, the ELISA kit was taken out from the refrigerator and allowed to equilibrate at room temperature for 1 h. PMMoV‐CP standard was diluted with the standard diluent buffer to obtain a series of standard dilutions (160, 80, 40, 20, 10 ng L^−1^). 50 µL of standard dilutions were added to the standard product well. 10 µL of sample, along with 40 µL of the sample diluent buffer, were added to the sample well. For the blank well, neither the sample nor the antibody was added, but all other steps remained the same. The plate was covered with a seal film and incubated at 37 °C for 30 min. After removing the seal film, the liquid was discarded and the plate were spun dry. Each well was then filled with the washing solution. After allowing it to stand for 30 s, the washing solution was discarded. The washing procedure was repeated five times. The plate was gently patted dry. 50 µL of horseradish peroxidase (HRP)‐labeled PMMoV antibody was added to each well and incubated at 37 °C for 30 min. The previously described washing procedure was then repeated. 50 µL coloring agent A and 50 µL coloring agent B were added to each well in turn. The plate was gently shaken and then incubate in the dark at 37 °C for 10 min. The blank well was used to zero the instrument before the measurement. After the addition of the stop solution, the absorbance at 450 nm was measured using a microplate reader (Infinite 200 PRO, TECAN, Switzerland).

### RT‐qPCR

First, total RNA was isolated from the filtrate with a TaKaRa MiniBEST Viral RNA/DNA Extraction Kit (TaKaRa, China) following the guidelines provided by the manufacturer. Second, the cDNA was synthesized from total RNA using a PrimeScriptTM reagent Kit with gDNA Eraser (Perfect Real Time) (TaKaRa, China) following the guidelines provided by the manufacturer. Subsequently, the reaction mixture was prepared with 2 µL of the cDNA template, 10 µL of TB Green Premix Ex Taq (Tli RnaseH Plus) (2×) (TaKaRa, code No. RR420A), 0.4 µL of forward primer (10 µmol L^−1^), 0.4 µL of reverse primer (10 µmol L^−1^), and 6.8 µL of nuclease‐free water. Amplification was conducted at 95 °C for 30 s (pre‐denaturation), 40 cycles of 95 °C for 5 s (denaturation), and 60 °C for 30 s (annealing/extension) in a ViiA7 real‐time PCR system (Applied Biosystems, USA). To generate standard curves, a series of ten‐fold dilutions (10‐10^7^ copies mL^−1^) of the synthesized standard plasmid that the target sequence (GenBank accession number MT904417.1) was cloned into vector pUC57 was prepared. The primer sequences were (5′‐3′) F: CAGTTGTAGGATTTTGCGGATTT; R: TTAGATTTCCTGCTACTGGTTTCAA.

### TEM

To investigate the morphological changes of PMMoV, 10 µL of PMMoV suspension was dropped onto 300‐mesh formvar/carbon‐coated molybdenum grids. The excess liquid was quickly absorbed by a filter paper. Subsequently, the grids were left to dry at room temperature and then observed by TEM (JEM‐2100, JEOL, Japan) at an accelerating voltage of 80 kV.

TEM was used to observe the morphology and size distribution of CP. 3 µL of the protein samples were dropped onto glow‐discharged 300‐mesh formvar/carbon‐coated copper grids for 40 s. The excess sample was absorbed by a filter paper. The grids were stained with 3 µL 3% uranyl acetate, and then washed twice with ultrapure water to remove the excess of uranyl acetate. Afterward, the grids were again stained with 3 µL 3% uranyl acetate for 40 s. The excess dye was absorbed by a filter paper. Finally, the grids were dried at room temperature before imaging. Micrographs were taken with a TEM (Tecnai G2 F20, FEI, USA) at an acceleration voltage of 200 kV.

### MST Assay

MST measurements were performed to detect the binding affinities of DOM with PMMoV particles and PMMoV‐CP using the Monolith NT.115 instrument (NanoTemper, Germany). A series of DOM stocks (≈30.5 nm to 1 mm) were prepared by two‐fold dilution in buffer1 (1×PBS, 0.05% Tween 20, pH 7.4). PMMoV particles and PMMoV‐CP was labeled with the fluorescent dye NT‐647 using the Monolith NT™ protein‐labeling kit RED‐NHS (NanoTemper, Germany) according to the manufacturer's protocol.

The labeled PMMoV particles and PMMoV‐CP (200 nm) was incubated with DOM stocks for 10 min in darkness at room temperature, and then loaded into standard capillaries. The measurements were carried out at 25 °C with 100% excitation power and 40% MST power. The MST data were analyzed using MO. Affinity analysis V2.3 software.

### Synthesis of Try‐Na

Try‐Na was synthesized by coordination reaction.^[^ ^54^ ^]^ First, NaOH (0.4 g, 10 mmol) and NaCl (0.58 g, 10 mmol) were dissolved in 20 mL of ultrapure water. Next, tryptophan (2.04 g, 10 mmol) was added to the solution and were stirred at room temperature for 6 h. After the reaction was completed, Try‐Na in solution was subsequently precipitated by adjusting the pH to 7. The prepared Try‐Na powder was filtered and washed with ultrapure water and finally dried at 40 °C for a week.

### LC‐MS

LC‐MS analysis was performed to detect decomposition products (Text S5, Supporting Information). Label‐free proteomics was employed to quantify oxidative damage in CP (Text S6, Supporting Information). PRM‐MS was used to quantify the target proteins in *Nicotiana benthamiana* (Text S7, Supporting Information).

### EPR

EPR was used to detect and quantify the radical species. 5,5‐dimethyl‐1‐pyrroline‐N‐oxide (DMPO) was used as the spin trapping agent for hydroxyl radical (·OH), carbon radical (·C), hydrogen radical (·H), and superoxide anion radical (·O_2_^‐^). 2,2,6,6‐tetramethyl‐4‐piperidone hydrochloride (TEMP) was used as the spin‐trapping agent for singlet oxygen (^1^O_2_).

### MD and QM/MM Simulations

MD simulations were performed using Desmond software. The system, consisting of 27795 atoms including 8442 water molecules (TIP3P), was subjected to an NPT ensemble at 300 K. The simulation duration was 105 ps, with the initial 5 ps designated for pre‐equilibration. The protein, comprising 157 residues and 2414 atoms, of which 1215 were heavy atoms. The simulation focused on elucidating protein‐ligand interactions, with key analyses including RMSD, RMSF, and secondary structure elements.

QM/MM calculations were conducted using the Qsite module. First, the structure of PMMoV‐CP was predicted by Alphafold. Afterward, the QM region was treated using the B3LYP level of theory with 6–31G(d) basis set. The remaining part of the system was described using the OPLS‐AA molecular mechanics force field. The QM/MM partitioning was carefully chosen to include key residues Try in the QM region, ensuring accurate modeling of the critical interactions.

### Statistical Analysis

The best subsets regression analysis was performed using the olsrr R package (version 0.5.3). This method compares all possible models and identifies the best‐fitting models that screen all possible combinations of variables. The optimal regression model was selected based on the minimum Akaike Information Criterion (AIC). Partial residual plots were applied to show the relationship between multiple predictors and the response. Z‐score method was used to standardized the data to improve the regression. Moreover, linear regression analysis was applied to analyze the relationship between environmental factors (turbidity, pH, dissolved oxygen, conductivity, and chloride) and the abundance of plant viruses using Origin software.

## Conflict of Interest

The authors declare no conflict of interest.

## Author Contributions

Y.W., M.C., and L.Y. designed the research. Y.W., M.C., L.Y., J.M., and J.T. performed research. Y.W., M.C., and L.Y. contributed new reagents/analytic tools. Y.W., M.C., L.Y., S.W., C.H., and J.P. analyzed data. Y.W., M.C., L.Y., J.M., C.H., and J.P.C. wrote the paper.

## Supporting information



Supporting Information

## Data Availability

The data that support the findings of this study are available from the corresponding author upon reasonable request.
