# Supplementary material for: Virome and Experimental Analysis Reveal Tryptophan‐Like Dissolved Organic Matter Contributes to the Persistence of Plant Viruses in River Water
Source: Adv Sci (Weinh). 2025 May 8;12(22):2417529. doi: 10.1002/advs.202417529 (PMC12165095; doi:10.1002/advs.202417529)
Supplement: Supplementary file 1 — Supporting Information [file ADVS-12-2417529-s001.docx]

Supporting Information

**Virome and Experimental Analysis Reveal Tryptophan-Like Dissolved Organic Matter Contributes to the Persistence of Plant Viruses in River Water**

*Yujie Wang^1,2^, Ming Chen^1,2*^, Liu Yang^1,2^, Jun Ma^1^, Jian Tang^1^, Shengjun Wu^1,2^, Chi He^3,*^, J. Paul Chen^4,5,*^*

^1^ Key Laboratory of Reservoir Aquatic Environment, Chongqing Institute of Green and Intelligent Technology, Chinese Academy of Sciences, Chongqing 400714, China.

^2^ Chongqing School, University of Chinese Academy of Sciences, Chongqing 400714, China.

^3^ State Key Laboratory of Multiphase Flow in Power Engineering, School of Energy and Power Engineering, Xi'an Jiaotong University, Xi'an 710049, Shaanxi, P.R. China.

^4^ Department of Civil and Environmental Engineering, National University of Singapore, 10 Kent Ridge, Singapore 117576, Singapore.

^5^ College of Chemistry and Environmental Engineering, Shenzhen University, Shenzhen 518060, P.R. China.

**Text S1. Diversity of RNA Viruses**

Following a rigorous set of viral annotation criteria (see Methods), we have successfully identified 1645 contigs that are attributed to RNA virus. These contigs were categorized into 46 families, 69 genera and 392 species. In almost all samples, several viral families were found to be highly abundant, accounting for 21.23%-99.99% of the total RNA viruses detected in water samples. These families included *Bromoviridae*, *Partitiviridae*, *Togaviridae*, *Flaviviridae*, *Pospiviroidae*, *Retroviridae*, *Marnaviridae*, *Dicistroviridae*, *Reoviridae*, *Potyviridae*, *Mitoviridae*, *Alphaflexiviridae*, *Totiviridae*, *Solspiviridae*, *Nodaviridae*, *Tospoviridae*, *Coronaviridae*, *Fiersviridae*, *Atkinsviridae*, and *Alphatetraviridae*, which were known to infect plants, fungi, protists and animals.

Among them, more than half of plant viruses are capable of infecting multiple host plants that includes several species from one or more different plant families (for example, alfalfa mosaic virus, cucumber green mottle mosaic virus, pepper mild mottle virus, tobacco mosaic virus, tomato mosaic virus, watermelon green mottle mosaic virus, youcai mosaic virus, papaya mosaic virus, apple chlorotic leaf spot virus, tobacco necrosis virus A, melon necrotic spot virus, cymbidium ringspot virus, hippeastrum chlorotic ringspot orthotospovirus, melon yellow spot orthotospovirus, tomato spotted wilt orthotospovirus, beet cryptic virus 2, raphanus sativus cryptic virus 2, papaya ringspot virus, sugarcane mosaic virus, watermelon mosaic virus, hop stunt viroid and citrus exocortis viroid). Compared to viruses associated with other host types, plant viruses were found to be dominant, counting 141972 reads belonging to the following families: *Bromoviridae*, *Endornaviridae*, *Virgaviridae*, *Alphaflexiviridae*, *Betaflexiviridae*, *Tombusviridae*, *Tospoviridae*, *Partitiviridae*, *Dicistroviridae*, *Marnaviridae*, *Potyviridae* and *Pospiviroidae*. There are other plant viruses that are currently known to only infect specific species of host plants (for example, members of *Endornaviridae*, *Virgaviridae*, *Alphaflexiviridae*, *Tombusviridae*, *Partitiviridae*, *Dicistroviridae* and *Marnaviridae*).

**Text S2.** **Water Quality Parameters**

The water quality parameters at each sampling site were measured with a portable multi-parameter water quality analyzer (ProQuatro, YSI, USA), such as dissolved oxygen, conductivity, pH, chloride. The turbidity of the water was measured by a portable turbidimeter (2100Q, HACH, USA). 50 mL of water sample were filtered by 0.45 μm membranes. All filtered water samples were stored at 4 °C in the dark until further DOM analysis. Three-dimensional excitation emission matrix (3D-EEM) fluorescence spectroscopy of water samples were measured by a fluorescence spectrophotometer (F-7000, Hitachi, Japan) for qualitative and quantitative evaluation of DOM. Filtered water samples was transferred into the four-sided transparent quartz cuvette. The range of excitation wavelength was set as 220-450 nm with an interval of 2 nm. The range of emission wavelength was set as 250-550 nm with an interval of 2 nm. The slit width of the excitation and emission were both set as 5 nm. The influence of Raman scattering and Rayleigh scattering were eliminated by subtracting the blank spectrum (ultrapure water) and interpolation method, respectively.^[1]^ 3D-EEM fluorescence spectral data were analyzed using fluorescence region integral (FRI) and fluorescence response percentage (Pi, n) methods.^[2]^ Each 3D-EEM fluorescence spectroscopy of DOM was divided into five fluorescence regions (Region I, tyrosine-like; Region II, tryptophan-like; Region III, fulvic acid-like; Region IV, soluble microbial by-product-like; and Region V, humic acid-like). The climate of this area is classified as a humid subtropical monsoon, characterized by hot and humid summers with abundant rainfall.^[3]^ The average annual water temperature is around 18 ℃, and the UV radiation levels reach approximately 9.5 mW/cm^2^.^[4, 5]^ The average pH value of the area is 8.^[6]^ Therefore, these conditions were chosen to simulate the natural aquatic environment.

**Text S3. Virome Approach**

For each sample, 10 L of water sample were collected, and then 1 mL of a 10 g/L solution of FeCl_3_ was added to facilitate the flocculation of viruses.^[7]^ After being incubated at room temperature for 1 h, the precipitate was filtered through 0.8 μm membranes of 90 mm diameter. Total nucleic acid was extracted from filtered membranes using the MiniBEST Viral RNA/DNA Extraction Kit Ver.5.0 (TaKaRa, China) according to the manufacturer's protocols. Subsequently, the amplification of whole transcriptome was performed using the Qiagen 150,054 REPLI-g Cell WGA & WTA Kit (Qiagen, Germany). Sequence libraries were generated using the ALFA-SEQ DNA Library Prep Kit for Illumina (FINDROP, Guangzhou) according to the manufacturer's protocols and then sequenced by Guangdong Magigene Biotechnology Co., Ltd. (Guangzhou, China) using the NovaSeq 6000 system (Illumina, USA) to yield 150 bp paired-end reads.

Raw reads were trimmed to remove adapter sequences and low-quality reads using SOAPnuke software (version 1.5.6) to obtain the clean reads.^[8]^ Subsequently, the clean reads were aligned to the ribosome database (Silva.132) and host database using BWA software (version 0.7.17).^[9]^ The alignment results that the alignment length was more than 80% of the total length of the reads were filtered. Next, the clean reads were assembled to contigs using MEGAHIT software (version 1.1.2). Additionally, BLASTN software (version 2.9.0+) was employed to compare the assembled contigs with the host sequence to identify and remove host sequences (proGenomes v.2.1 database).^[10]^ CD-HIT software (version 4.7) was used to cluster the contigs with a 95% sequence identity.

The complementary approaches were applied to detect viral sequences. The first approach utilized for virus identification relied on known viral sequences. The presence of viral sequences was confirmed by BLAST searches on non-redundant contigs against the virus-NT database, which consists of viruses isolated from the NT database. Non-redundant contigs that met the criteria of alignment similarity of at least 80%, alignment length of at least 500 bp, and e-value≤1×10^-5^ were defined as viral sequences. In order to identify unknown viruses and reduce the rate of false positives, the second approach was employed, which involved inferring viral sequences based on references from multiple databases.^[11]^ Firstly, candidate viral sequences were identified by satisfying at least one of the following three conditions: (1) non-redundant contigs were searched against the Virus-NT database using BLASTN software with an e-value≤1×10^-5^; (2) non-redundant contigs were searched against the Virus-NR database that isolated from NR database using BLASTX software (version 2.9.0+) with an e-value≤1×10^-3^; (3) genes in non-redundant contigs were predicted using MetaGeneMark software (version 3.38), and then the protein sequences were compared with virus protein family databases (VPFs and vFam) using hmmsearch software (version 3.2.1) with an e-value≤1×10^-5^.^[12]^ To eliminate false positives, the obtained candidate viral sequences were subjected to a BLAST search against the NT database to screen the alignment results using a cutoff of e-value≤1×10^-10^. For the obtained candidate viral sequences that did not align in the previous step, DIAMOND software (version 0.9.10) was used to align sequences against the NR database to screen the alignment results using a cutoff of e-value≤1×10^-3^. The screened sequences were annotated using the NCBI taxonomy database. If more than 20% of the top 50 alignment results were classified as non-viral sequences (such as Eukaryota, Bacteria, Archaea), these sequences were considered as non-viral sequences. Otherwise, the remaining sequences were considered as viral sequences. The viral sequences were annotated by the best BLAST hit against the virus-NT database (length ≥ 500 bp and e-value≤1×10^-5^). The annotation of DNA viruses sequences by both methods were removed. The viral sequences obtained through the aforementioned two methods underwent further screening, where the sequences annotated as DNA viruses were excluded, while the sequences annotated as RNA viruses were retained. To calculate the relative abundance of RNA viruses, the clean reads after host removal were aligned against viral contigs using BWA software (version 0.7.17). The alignment results with length shorter than 80% of the total length of reads were filtered out, and the number of viral reads were counted. RPKM (reads per kilobase of contig sequence per million reads) values were calculated for all viral contigs to estimate the abundance of RNA viruses.

**Text S4. Expression and Purification of TSWV-CP and PRSV-CP**

To express TSWV-CP and PRSV-CP, the corresponding genes (GenBank accession No. JF960235.1, bases 2043-2819; GenBank accession No. MG564513.1) was cloned into a pET28a vector, respectively. The TSWV-CP gene were added a PreScission protease cleavage site and a 6 × His tag sequence at the N-terminus. The PRSV-CP gene were added a 6 × His tag sequence and a tobacco etch virus (TEV) protease cleavage site at the C-terminus. The recombinant plasmid was then transformed into expression host strain E. coli Rosetta(DE3) pLysS by heat shock treatment. Cells carrying the recombinant plasmid were grown in LB liquid medium containing 50 μg/mL kanamycin at 37 °C with shaking (220 rpm) until reaching OD600 = 0.6-0.8. The protein expression was induced by adding IPTG with a final concentration of 0.2 mM overnight at 18 °C. The cells were collected with centrifugation at 4,000 rpm for 15 min at 4 °C and used for subsequent purification.

For TSWV-CP purification, the collected cells were mixed with Ni-Buffer A (50 mM NaH2PO4, 300 mM NaCl, 1 mM TCEP, 0.5 mM PMSF, 10 μg/mL DNase I, 10% glycreol, pH 7.4) at a ratio of 1:10 (1 g cells/10 mL buffer) and lysed by sonication in ice bath. Lysates were collected by centrifugation at 12,000 rpm for 90 min at 4 °C, loaded on Ni-bead columns (15 mL) equilibrated with Ni-Buffer A, washed with Ni-Buffer B (50 mM NaH2PO4, 300 mM NaCl, 1 mM TCEP, 10% glycreol, 20 mM imidazole, pH 7.4) and eluted using Ni-Buffer C (50 mM NaH2PO4, 300 mM NaCl, 1 mM TCEP, 10% glycreol, 250 mM imidazole, pH 7.4). The purified protein was cleaved by PreScission protease and loaded on Ni-bead columns (10 mL) equilibrated with Ni-Buffer A (50 mM NaH2PO4, 300 mM NaCl, 1 mM TCEP, 5% glycreol, pH 7.4). The columns were washed with Ni-Buffer B (50 mM NaH2PO4, 300 mM NaCl, 1 mM TCEP, 5% glycreol, 20 mM imidazole, pH 7.4) and Ni-Buffer C (50 mM NaH2PO4, 300 mM NaCl, 1 mM TCEP, 5% glycreol, 250 mM imidazole, pH 7.4). The obtained proteins were diluted with buffer (20 mM Tris-HCl, 1mM TCEP, pH 7.4) to a final NaCl concentration of 100 mM, further purified using a HiTrap Q HP column (5 mL) equilibrated with Q-Buffer A (20 mM Tris-HCl, 100 mM NaCl, 1mM TCEP, pH 7.4), washed with Q-Buffer A and eluted with a linear gradient of 0–100% Q-Buffer B (20 mM Tris-HCl, 1M NaCl, ,1mM TCEP, pH 7.4). The high purity of TSWV-CP (>90%) was collected and stored at −80 °C.

For PRSV-CP purification, the collected cells were mixed with Ni-Buffer A (50 mM Tris, 300 mM NaCl, 1 mM TCEP, 10 μg/mL DNase I, 5% glycerol, pH 8) at a ratio of 1:10 (1 g cells per 10 mL buffer) and then lysed by sonication in an ice bath. Lysates were collected by centrifugation at 12,000 rpm for 90 min at 4 °C, loaded on Ni-bead columns (5 mL) equilibrated with Ni-Buffer A. The columns were washed with Ni-Buffer B (50 mM Tris, 300 mM NaCl, 1 mM TCEP, 5% glycreol, 20 mM imidazole, pH 8) and eluted using Ni-Buffer C (50 mM Tris, 300 mM NaCl, 1 mM TCEP, 5% glycreol, 300 mM imidazole, pH 8). The purified protein was cleaved by TEV protease and loaded on Ni-bead columns (5 mL) equilibrated with Ni-Buffer A (50 mM Tris, 300 mM NaCl, 1 mM TCEP, 5% glycreol, pH 8). The columns were washed with Ni-Buffer B (50 mM Tris, 300 mM NaCl, 1 mM TCEP, 5% glycreol, 20 mM imidazole, pH 8) and eluted using Ni-Buffer C (50 mM Tris, 300 mM NaCl, 1 mM TCEP, 5% glycreol, 300 mM imidazole, pH 8). The obtained proteins were diluted with buffer (50 mM Tris-HCl, 1mM TCEP, 5% glycreol, pH 8) to a final NaCl concentration of 100 mM, further purified using a HiTrap Q HP column (5 mL) equilibrated with Q-Buffer A (50 mM Tris-HCl, 100 mM NaCl, 1mM TCEP, 5% glycreol, pH 8), washed with Q-Buffer A and eluted with a linear gradient of 0–100% Q-Buffer B (50 mM Tris-HCl, 1M NaCl, 1mM TCEP, 5% glycreol, pH 8). The high purity of PRSV-CP (>90%) was collected and stored at −80 °C.

**Text S5. LC-MS**

The liquid sample was filtered using a 0.22 μm membrane and then analyzed by LC-MS (Agilent 1290-6530, Agilent, USA) with ZORBAX Eclipse Plus C18 column (2.1 × 50.0 mm, 1.8 µm, Agilent). The mobile phases consisted of methanol (A) and 0.1% formic acid in water (B), following gradient: 0-2 min (A:B, 1:9); 10-13 min (A:B, 9:1), 13.5-16 min (A:B, 1:9). The flow rate was 0.3 mL/min, and the column temperature was 40 °C. MS analysis was performed in positive electrospray ionization (ESI) mode with the m/z range of 100-3200 m/z.

**Text S6. Lable-free proteome**

Label-free proteome was applied to evaluate the effects of various treatments on the primary structure of PMMoV-CP. According to the filter-aided method, 900 µL of sample from each group was subjected to digestion with trypsin and Glu-C.^[13]^ The digest peptides of each group were desalted on C18 Cartridges (Empore™ SPE Cartridges C18, Sigma, USA). After desalting, the peptides were concentrated using vacuum centrifugation and reconstituted in 40 µL of 0.1% (v/v) formic acid.

Peptides were analyzed on a nano-flow ultra-high pressure liquid chromatography system (nanoElute, Bruker, Germany) coupled to a trapped ion mobility-quadrupole time-of-flight mass spectrometer (timsTOF Pro, Bruker, Germany). Peptides were injected onto the C18 reversed-phase analytical column with the mobile phase of 0.1 % formic acid solution and eluted at a constant flow rate of 300 nL/min with a linear gradient of acetonitrile/formic acid (99.9/0.1, v/v). The mass spectrometer was operated in positive polarity mode in the range of m/z 100–1700 with a captive spray ion source run at 1.5 kV.

Data acquisition mode was used in parallel accumulated serial fragmentation (PASEF) mode with the following settings: MS/MS PASEF scans per cycle, 10; ion mobility coefficients (1/K_0_), 0.6-1.6 Vs/cm^2^; active exclusion time, 24 s. The ratio of carbonylation for each site in proline and arginine were calculated using MaxQuant (version 1.6.14) software by comparing the intensity of modified peptides to their corresponding detected peptides.^[14]^

**Text S7. PRM-MS**

To quantify the target proteins, PRM-MS was performed using mass spectrometer (Q-Exactive HF-X, Thermo Scientific, USA) coupled to a nanoflow liquid chromatograph (EASY-nLC 1200, Thermo Scientific, USA). Total leaf proteins were extracted using a modified Borax/PVPP/Phenol (BPP) protocol as described.^[15]^ The extracted proteins were then subjected to automated reduction, alkylation, digestion.

The digest peptides were detected by liquid chromatography (EASY-nLC 1200, Thermo, USA) with a C18 column (75 μm × 25 cm, Thermo, USA) coupled to mass spectrometry (Q-Exactive HF-X, Thermo, USA). The mobile phase A (0.1% formic acid in 2% acetonitrile) and mobile phase B (0.1% formic acid in 80% acetonitrile) at a flow rate of 300 nL/min were utilized for elution following gradient: 0-64 min, 5-23 % B; 64-80 min, 23-29 % B; 80-90 min, 29-38 % B; 90-92 min, 38-48 % B; 92-93 min, 48-100 % B; 93-120 min, 100 % B.

The mass spectrometer was operated in PRM mode using high energy collision induced dissociation fragmentation methods (normalized collision energy of 28%). The full scan MS was conducted with the following settings: scanning range, 300-1500 m/z; resolution, 60,000; AGC target, 3×10^6^; maximum IT, 20 ms. The MS2 scans were set with resolution of 15,000, AGC target of 5×10^5^ and maximum IT of 80 ms. The original mass spectrum data were imported into Skyline software to obtain the retention time of the target peptide and the peak area of each peptide. This information was used to confirm the target proteins and analyze their relative expression levels.


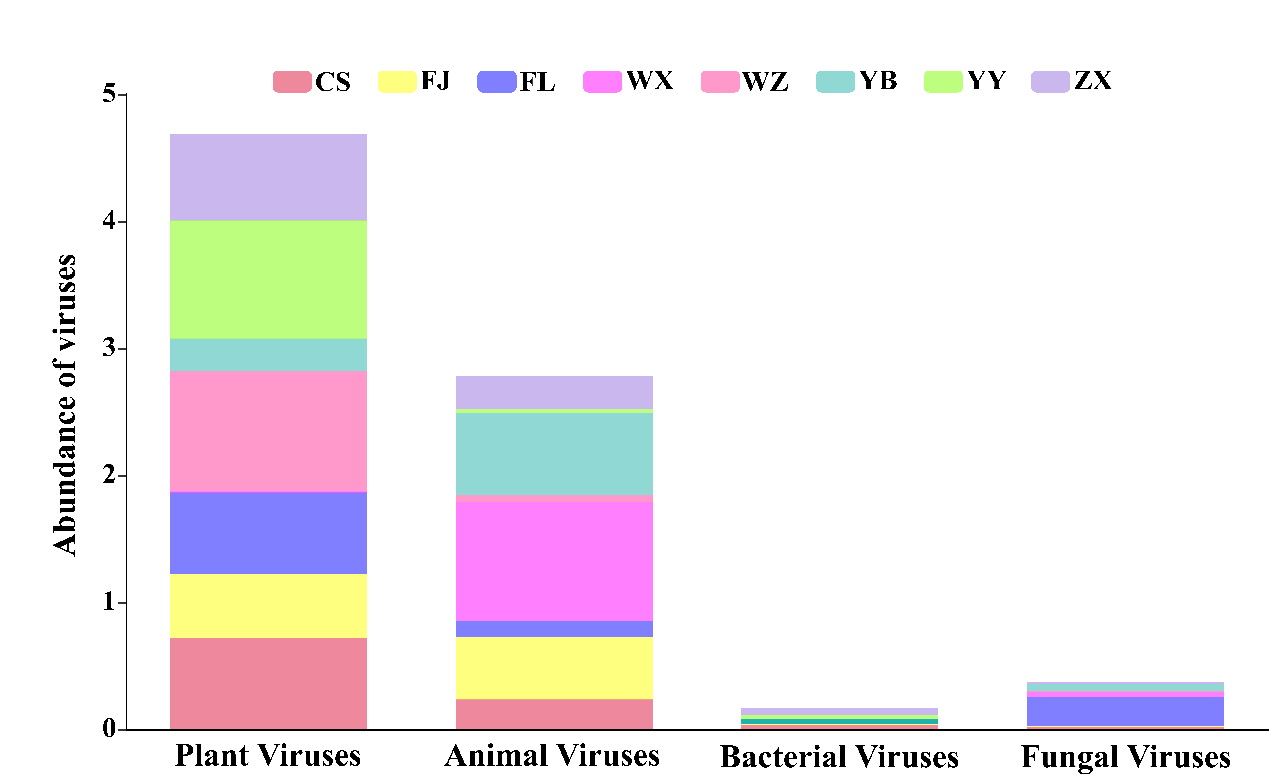


**Figure S1.** The abundance of viruses with different host types across sampling sites.


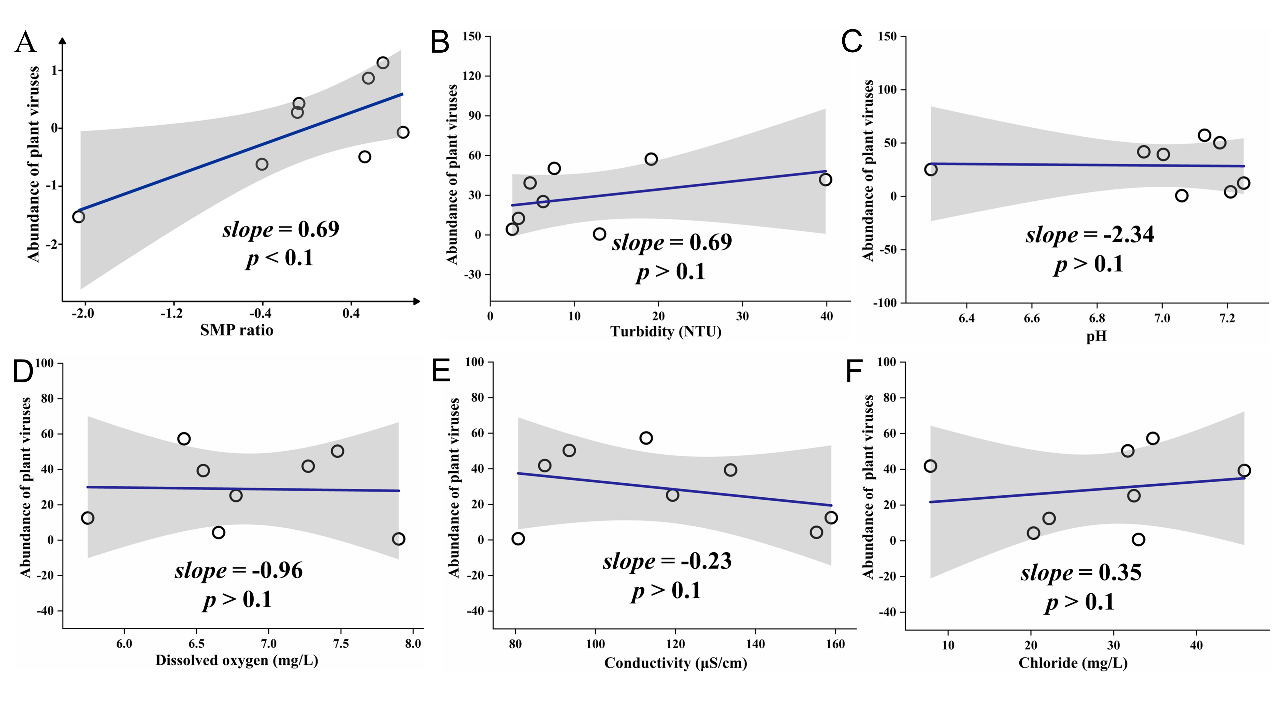


**Figure S2.** The correlation between environmental factors and the abundance of plant viruses in river water. A) The partial residual plot between the abundance of plant viruses and SMP ratio. B) The linear regression plot between the abundance of plant viruses and turbidity. C) The linear regression plot between the abundance of plant viruses and pH. D) The linear regression plot between the abundance of plant viruses and dissolved oxygen. E) The linear regression plot between the abundance of plant viruses and conductivity. F) The linear regression plot between the abundance of plant viruses and chloride.


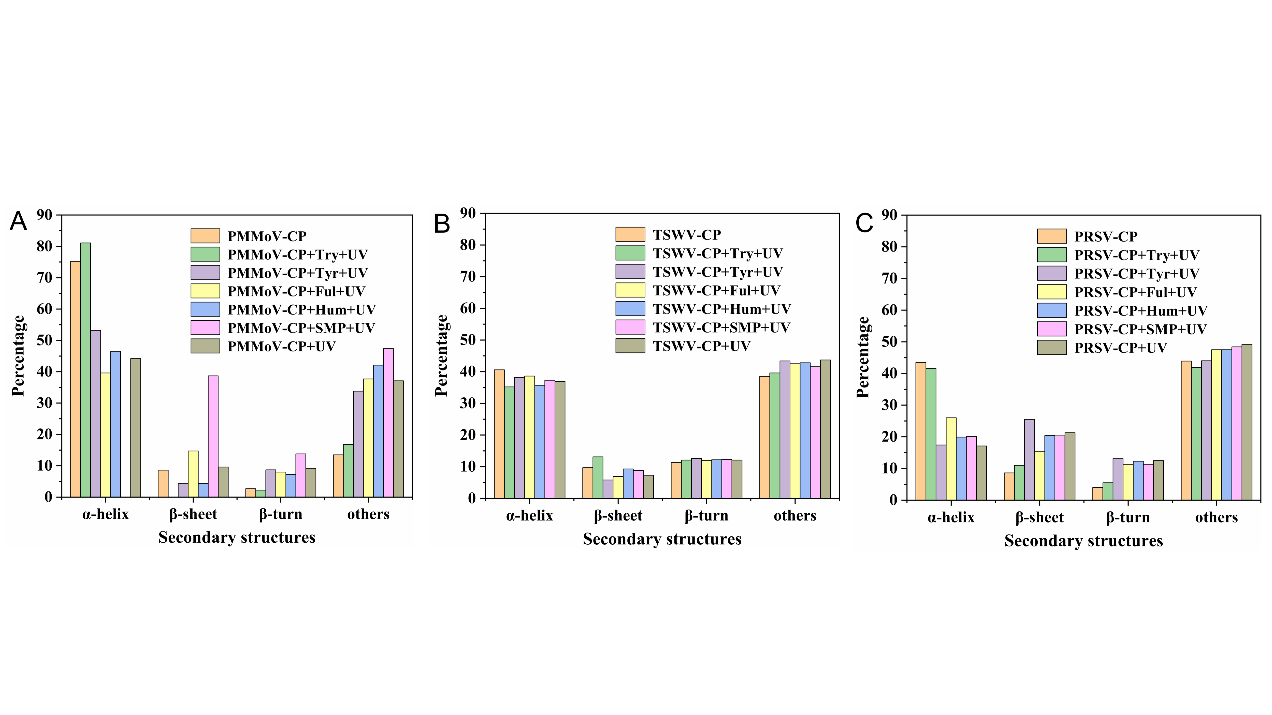


**Figure S3.** Comparison of secondary structure content in PMMoV-CP, TSWV-CP and PRSV-CP without and with DOM after UV radiation. A) The secondary structure content of PMMoV-CP without and with DOM (Try, Tyr, Ful, Hum and SMP) after UV radiation. B) The secondary structure content of TSWV-CP without and with DOM (Try, Tyr, Ful, Hum and SMP) after UV radiation. C) The secondary structure content of PRSV-CP without and with DOM (Try, Tyr, Ful, Hum and SMP) after UV radiation.


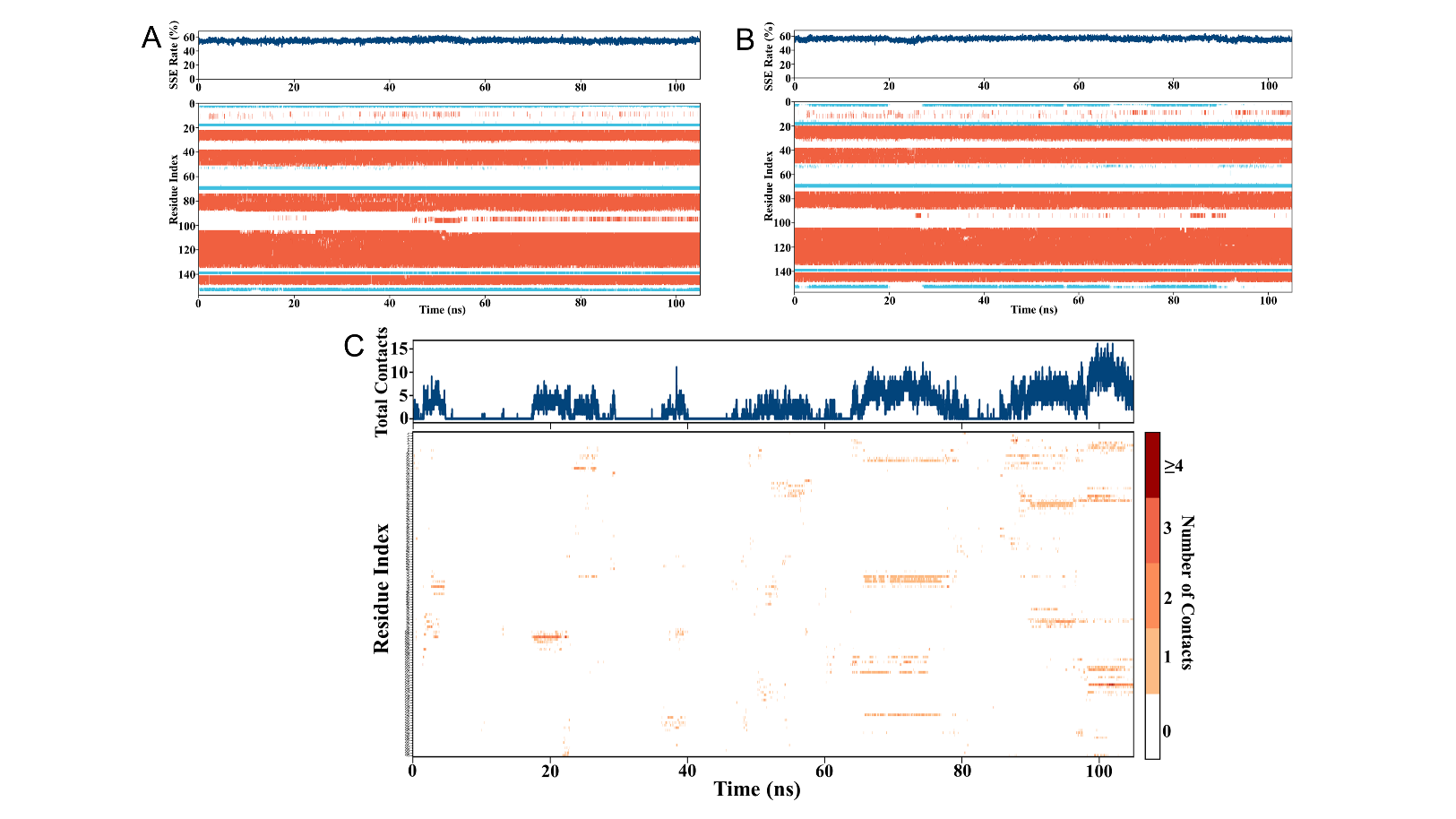


**Figure S4.** MD simulations of PMMoV-CP without and with Try. A) The plot above summarizes the secondary structure elements (SSE) of PMMoV-CP in water. The plot below tracks the SSE assignment of each residue over time in water, with α-helices shown in red and β-sheets in blue. B) The plot above summarizes the secondary structure elements (SSE) of PMMoV-CP in Try solution. The plot below tracks the SSE assignment of each residue over time in Try, with α-helices shown in red and β-sheets in blue. C) The plot above shows the total number of contacts that PMMoV-CP makes with Try throughout the trajectory. The plot below illustrates the residues interacting with Try in each frame of the trajectory. The intensity of the contacts is denoted by shades of orange, as per the scale located to the right of the plot.

**
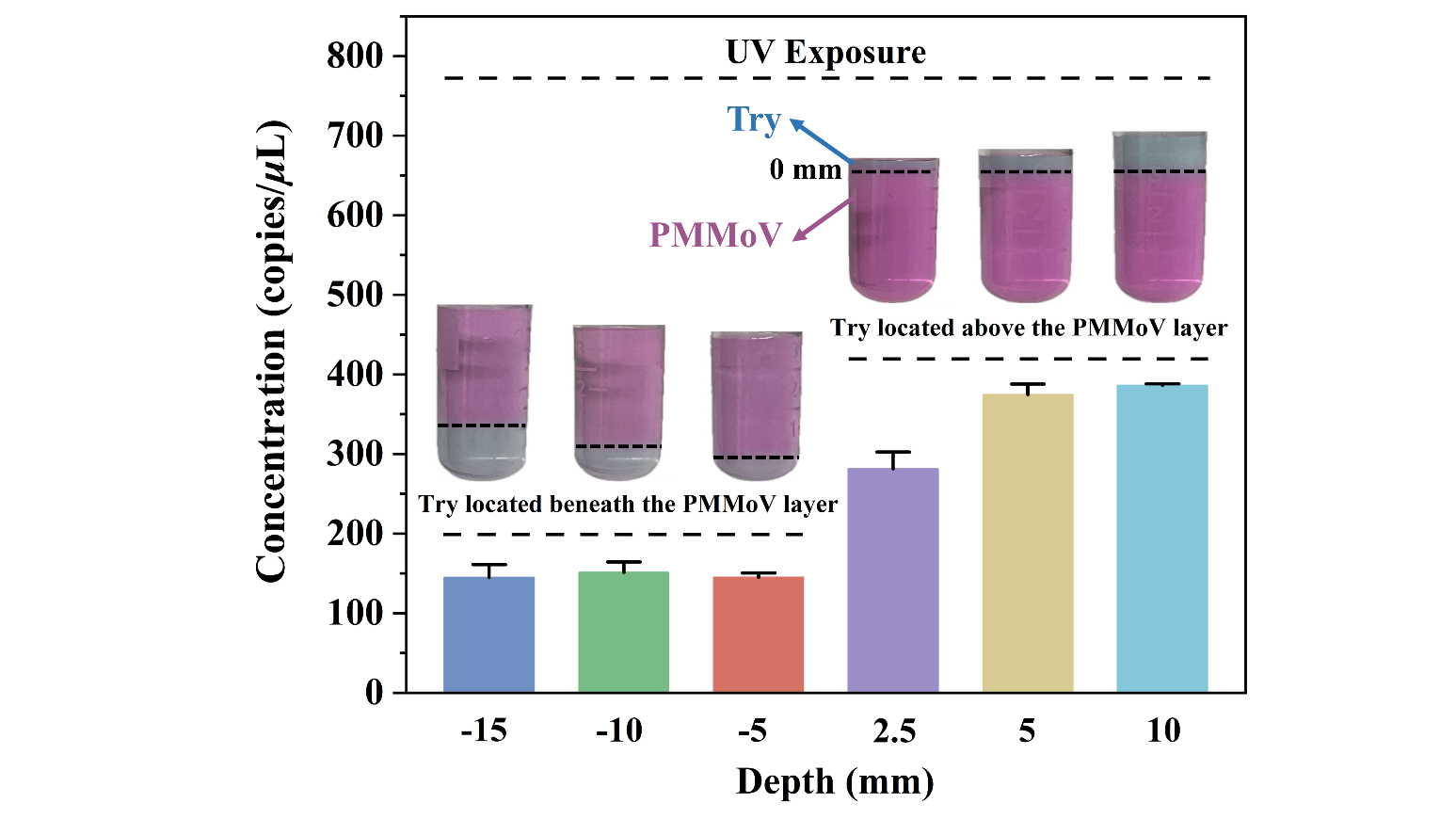
**

**Figure S5.** RT-qPCR measurement of PMMoV-CP gene levels in PMMoV (shown in red), with varying depths of the Try layer (shown in blue), after 12 h under UV radiation. Left: Try located beneath the PMMoV layer; Right: Try located above the PMMoV layer.

**
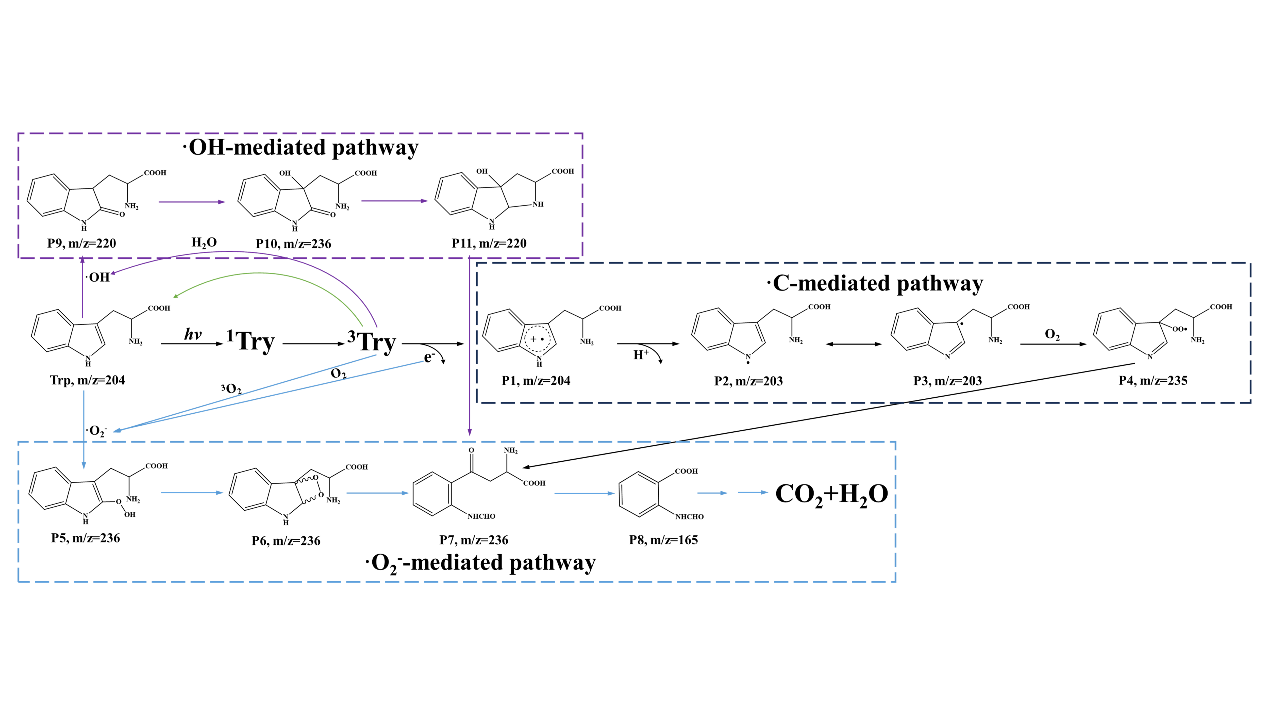
**

**Figure S6.** Schematic diagram illustrating the key reaction pathways of Try in “swimming firewall mode”.


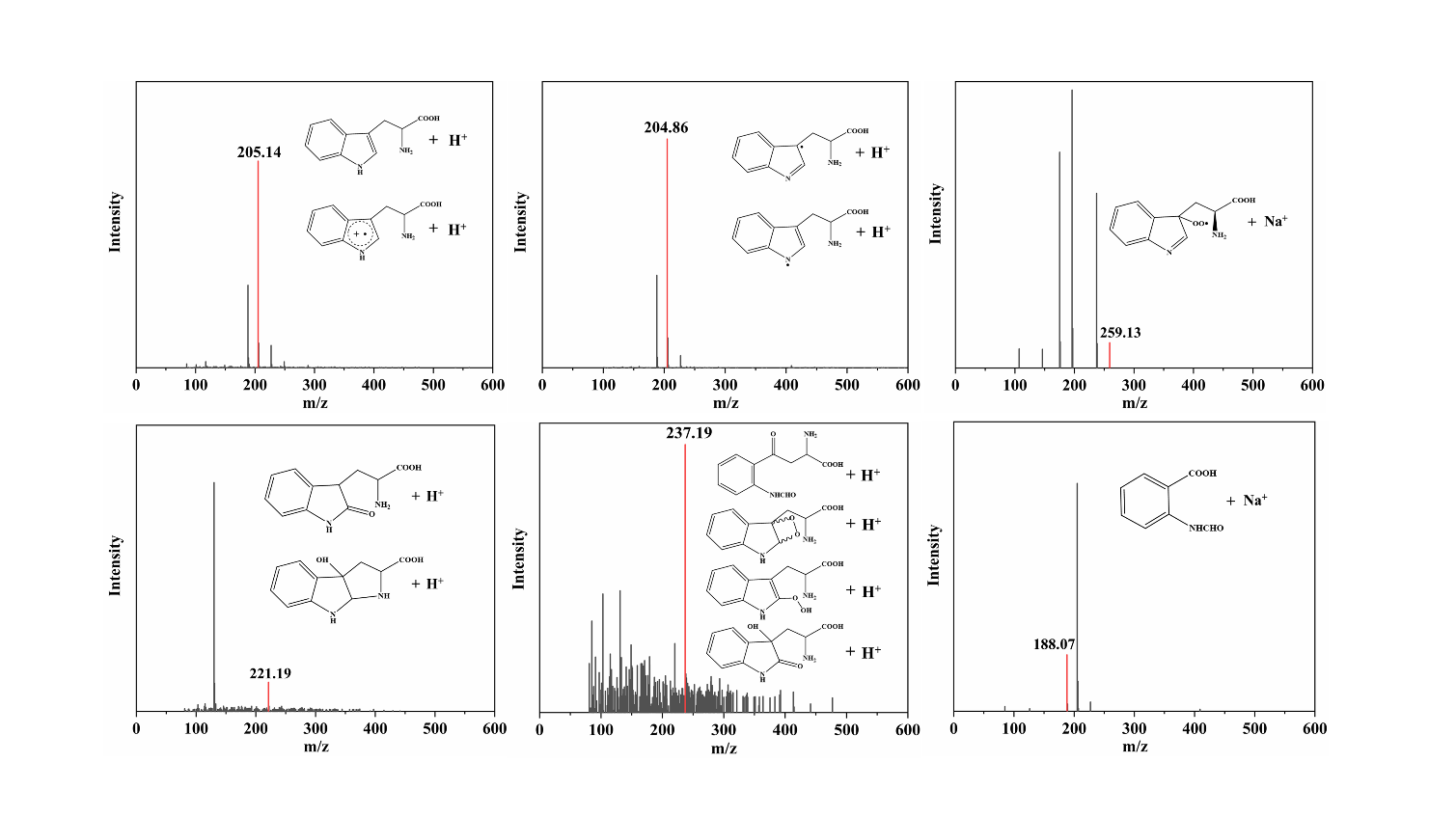


**Figure S7.** Decomposition products from Try in “swimming firewall mode”.


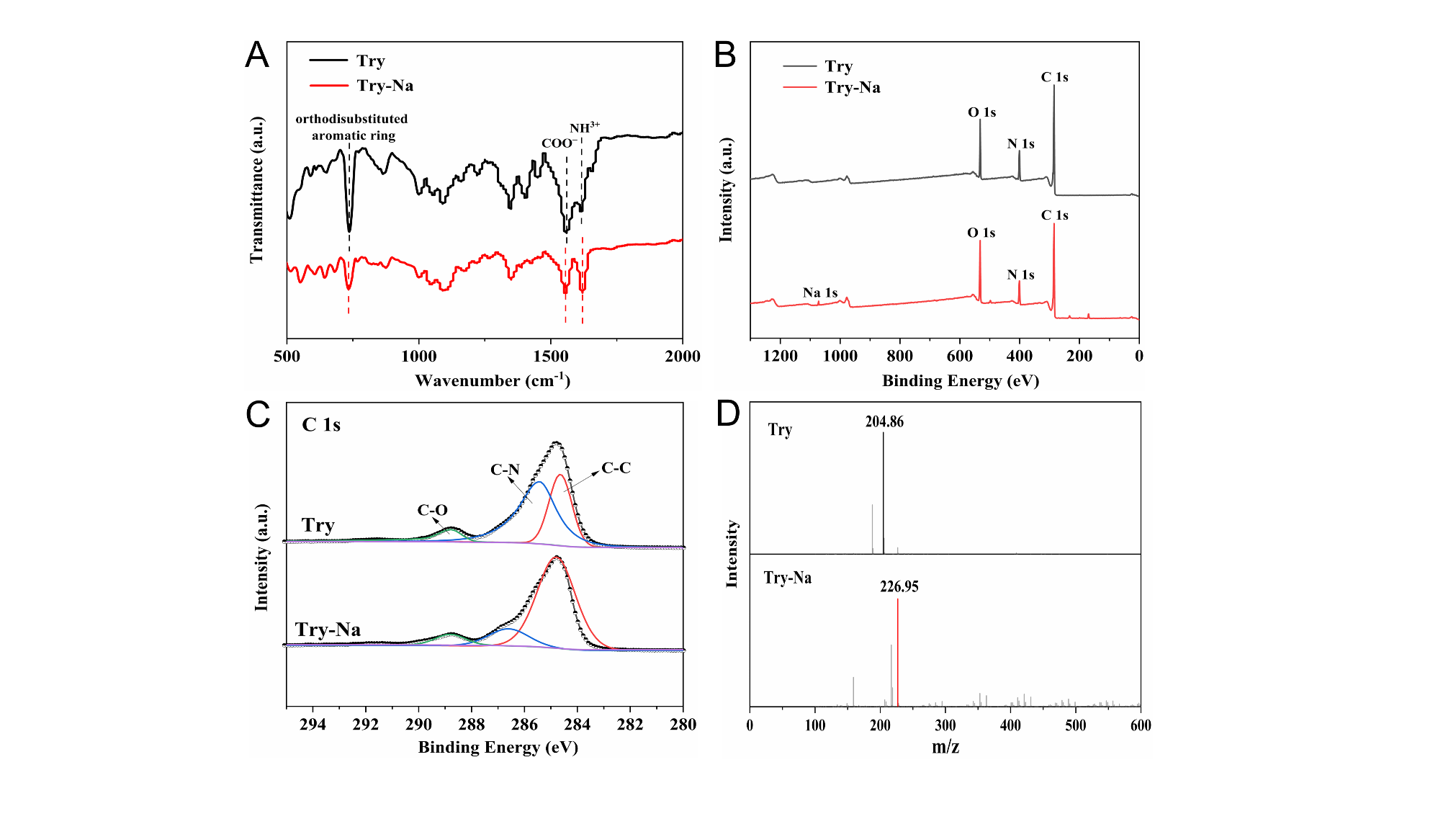


**Figure S8.** Characterization of Try-Na and Try through several analytical techniques. A) FTIR spectra of Try and Try-Na. B) XPS survey spectra of Try and Try-Na. C) High-resolution XPS spectra of Try and Try-Na. D) LC-MS diagram of Try and Try-Na.


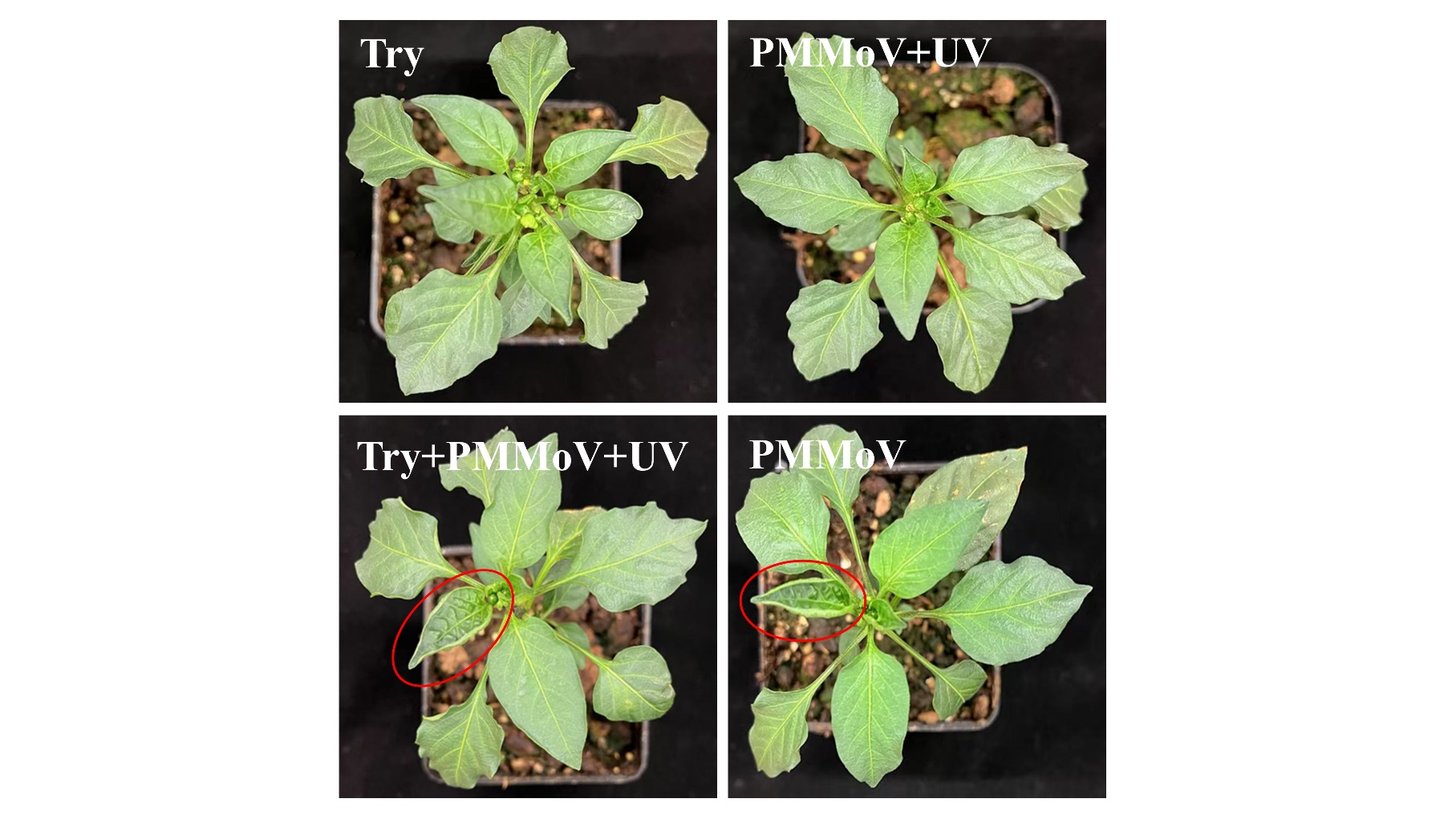


**Figure S9.** Symptoms of *Capsicum annuum L*. inoculating Try, untreated PMMoV, PMMoV and PMMoV with Try after UV radiated. Photographs were taken at 10 days post-inoculation (dpi).


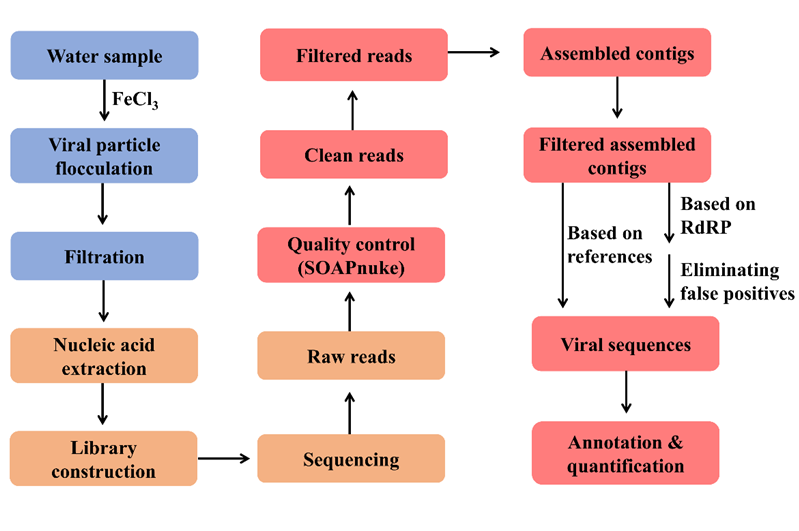


**Figure S10.** Workflow for virome analysis from river samples.

**Table S1.** The detailed sequence information of samples.

| Sample | Raw base (G) | Clean reads | Virus reads | Contigs |
| --- | --- | --- | --- | --- |
| YB1 | 11.7 | 30986275 | 26082 | 202257 |
| YB2 | 11.7 | 31071073 | 20006 | 169604 |
| CS1 | 12.5 | 23555501 | 13302 | 87751 |
| CS2 | 11.6 | 22023374 | 14778 | 90471 |
| FL1 | 10 | 26874269 | 46423 | 106553 |
| FL2 | 11 | 29206266 | 75124 | 22038 |
| ZX1 | 10.8 | 28493811 | 17017 | 67257 |
| ZX2 | 10.8 | 28242443 | 19937 | 16617 |
| WZ1 | 12.7 | 32949679 | 14598 | 66252 |
| WZ2 | 11.1 | 29579866 | 8751 | 57023 |
| YY1 | 10.7 | 28485336 | 15606 | 96982 |
| YY2 | 10.8 | 29168124 | 14788 | 47735 |
| FJ1 | 9.9 | 18624477 | 13812 | 97506 |
| FJ2 | 10.6 | 17147099 | 23232 | 86059 |
| WS1 | 12.6 | 32453579 | 34689 | 23495 |
| WS2 | 10.8 | 26156944 | 58836 | 11239 |

**Table S2.** Analysis of best subset regression for determining the best-fit model.

| Model | C(p) | AIC | SBIC | SBC | MSEP | FPE | HSP | APC |
| --- | --- | --- | --- | --- | --- | --- | --- | --- |
| 1 | 1.6791 | 25.05 | 3.6476 | 25.2883 | 6.8409 | 1.0557 | 0.1689 | 1.2065 |
| 2 | 0.5841 | 20.7507 | 1.8377 | 21.0685 | 3.891 | 0.6341 | 0.1153 | 0.7247 |
| 3 | 2.1227 | 21.1771 | 5.292 | 21.5744 | 4.2616 | 0.7103 | 0.1578 | 0.8117 |
| 4 | 4.0103 | 22.7418 | 12.0788 | 23.2185 | 6.0538 | 0.9716 | 0.299 | 1.1104 |
| 5 | 6 | 24.7008 | 19.9978 | 25.2569 | 12.0457 | 1.5615 | 0.8923 | 1.7846 |

Note: * The best model for the selection criterion is according to the minimum Akaike Information Criterion (AIC). Variables in model 1: humic-like DOM; variables in model 2: tryptophan-like DOM and soluble microbial products; variables in model 3: fulvic-like DOM, humic-like DOM and soluble microbial products; variables in model 4: tyrosine-like DOM, tryptophan-like DOM, fulvic-like DOM and soluble microbial products; variables in model 5: tyrosine-like DOM, tryptophan-like DOM, fulvic-like DOM, humic-like DOM and soluble microbial products.

**SI References**

[1] M. Bahram, R. Bro, C. Stedmon, A. Afkhami, *J. Chemom.* **2006**, 20, 99.

[2] W. Chen, P. Westerhoff, J. A. Leenheer, K. Booksh, *Environ. Sci. Technol.* **2003**, 37, 5701.

[3] S. Zhang, L. Ye, C. Huang, M. Wang, Y. Yang, T. Wang, W. Tan, *Catena* **2022**, 219, 106601.

[4] W. Guo, N. He, G. Dou, J. Hu, H. Wang, *Int. J. Environ. Res. Public Health* **2021**, 18, 12039.

[5] C. Hu, D. Chen, J. Liao, *J. Meteorol. Environ.* **2009**, 25, 23.

[6] Y. Huang, T. Pan, X. Yang, X. Fan, M. Guo, *J. Henan Norm. Univ.* **2022**, 50, 71.

[7] S. G. John, C. B. Mendez, L. Deng, B. Poulos, A. K. M. Kauffman, S. Kern, J. Brum, M. F. Polz, E. A. Boyle, M. B. Sullivan, *Environ. Microbiol. Rep.* **2011**, 3, 195.

[8] Y. Chen, Y. Chen, C. Shi, Z. Huang, Y. Zhang, S. Li, Y. Li, J. Ye, C. Yu, Z. Li, *Gigascience* **2018**, 7, 1.

[9] H. Li, R. Durbin, *Bioinformatics* **2009**, 25, 1754.

[10] D. R. Mende, I. Letunic, O. M. Maistrenko, T. S. Schmidt, A. Milanese, L. Paoli, A. Hernández-Plaza, A. N. Orakov, S. K. Forslund, S. Sunagawa, *Nucleic Acids Res.* **2020**, 48, D621.

[11] G. Zhao, G. Wu, E. S. Lim, L. Droit, S. Krishnamurthy, D. H. Barouch, H. W. Virgin, D. Wang, *Virology* **2017**, 503, 21.

[12] D. Paez-Espino, E. A. Eloe-Fadrosh, G. A. Pavlopoulos, A. D. Thomas, M. Huntemann, N. Mikhailova, E. Rubin, N. N. Ivanova, N. C. Kyrpides, *Nature* **2016**, 536, 425.

[13] J. R. Wiśniewski, *Anal. Chem.* **2016**, 88, 5438.

[14] S. Tyanova, T. Temu, J. Cox, *Nat. Protoc.* **2016**, 11, 2301.

[15] C. Wang, L. Chen, Z. c. Cai, C. Chen, Z. Liu, X. Liu, L. Zou, J. Chen, M. Tan, L. Wei, *J. Agric. Food Chem.* **2020**, 68, 1480.
